# Nanodefensin-encased hydrogel with dual bactericidal and pro-regenerative functions for advanced wound therapy

**DOI:** 10.7150/thno.53089

**Published:** 2021-01-26

**Authors:** Gan Luo, Yaqi Sun, Jue Zhang, Zhipeng Xu, Wuyuan Lu, Hanbin Wang, Yan Zhang, Hui Li, Zhengwei Mao, Shixin Ye, Baoli Cheng, Xiangming Fang

**Affiliations:** 1 Department of Anesthesiology and Intensive Care, The First Affiliated Hospital, School of Medicine, Zhejiang University, Hangzhou 310003, China.; 2 Department of Polymer Science and Engineering, Zhejiang University, Hangzhou 310027, China.; 3 Laboratory of Computational and Quantitative Biology (LCQB), Institute of Biology, Paris-Seine, Sorbonne University, Paris, 75006, France.; 4 Key Laboratory of Medical Molecular Virology (MOE/NHC/CAMS), School of Basic Medical Sciences, Fudan University, Shanghai, 200032, China.

**Keywords:** host defense peptides, wound healing, regenerative medicine, biomaterials, pharmaceutical formulation

## Abstract

**Background:** Host defense peptides (HDPs) have emerged as a novel therapeutic paradigm for wound management; however, their clinical applications remain a challenge owing to their poor pharmacological properties and lack of suitable pharmaceutical formulations. Nanodefensin (ND), a nanoengineered human α-defensin 5 (HD5), has shown improved pharmacological properties relative to the parent compound. In this study, we engineered a nanodefensin-encased hydrogel (NDEFgel), investigated the effects of NDEFgel on wound healing, and elucidated underlying mechanisms.

**Method:** ND was chemically synthesized and tested functions by* in vitro* antimicrobial and scratch assays and western blotting. Different NDEFgels were evaluated by* in vitro* characterizations including degradation, drug release and antimicrobial activity. In full-thickness excisional murine models, the optimal NDEFgel was directly applied onto wound sites, and the efficacy was assessed. Moreover, the underlying mechanisms of pro-regenerative effect developed by NDEFgel were also explored.

**Results:** Apart from bactericidal effects, ND modulated fibroblast behaviors by promoting migration and differentiation. Among the tested hydrogels, the Pluronic F127 (Plu) hydrogel represented the most desirable carrier for ND delivery owing to its favorable controlled release and compatibility with ND. Local treatment of NDEFgel on the wound bed resulted in accelerated wound regeneration and attenuated bacterial burden. We further demonstrated that NDEFgel therapy significantly upregulated genes related to collagen deposition and fibroblasts, and increased the expression of myofibroblasts and Rac1. We therefore found that Rac1 is a critical factor in the ND-induced modulation of fibroblast behaviors *in vitro* through a Rac1-dependent cytoskeletal rearrangement.

**Conclusion:** Our results indicate that NDEFgel may be a promising dual-action therapeutic option for advanced wound management in the future.

## Introduction

The skin is the largest body organ, functioning as a barrier to prevent pathogens from invading the body. A variety of wounds, including burns, acute postsurgical wounds, and chronic ulcers, dramatically threaten human life and burden public health 1, and potentially become one of the leading causes of death worldwide 2, 3. Thus, employing therapeutic agents to control wounds rapidly and effectively is very important for trauma emergencies. Although current therapeutic options (*e.g.,* Prontosan and dermagraft) are relatively effective in the clinic 4, 5, more adaptive and/or multifunctional approaches are still being developed to advance wound management.

Host defense peptide (HDP)-based therapies have opened a new avenue for wound treatment and have shown promising ability in promoting wound healing 6, 7. Complex pathophysiological events such as intestinal homeostasis, wound healing, and host immune responses, usually require the participation of HDPs 8-11, such as cathelicidin LL-37 12, hepcidin 13, neutrophil-derived α-defensin 1-4, and enteric α-defensin 5 and 6 7, 8, 14, 15. Increasing investigations have uncovered the potential therapeutic roles of HDPs in wound healing 7, 9, 10, 16. Human β-defensin 3 responsive to skin injury could be secreted to participate in innate immunity during wound healing 10. Epithelial hepcidin, capable of recruiting neutrophils, contributes to attenuated bacterial infections during tissue injury 16. Thrombin-derived HDP, TCP-25, can kill pathogens and neutralize endotoxins and has been engineered to be a dual-action hydrogel targeting both wound infection and inflammation 9.

Human α-defensin 5 (HD5), an enteric HDP expressed by Paneth cells, exhibits broad-spectrum activity against both gram-negative and gram-positive bacteria 15, 17 and has been found to mediate cell migration and proliferation *in vitro* 18; thus, HD5 can be considered another dual-action therapeutic for advanced wound care. However, HD5 faces some therapeutic challenges, such as poor physicochemical stability and insufficient activity 19, 20, that severely limit the clinical translation of HD5 as a wound-healing agent. Previously, we demonstrated that C-terminal myristoylation of HD5 via a Gly-Lys linker could significantly improve its biological activity and physicochemical stability by achieving supramolecular nanoassembling behaviors 20; thus, the resultant nanodefensin (ND) shows improved therapeutic performance in sepsis. However, the therapeutic efficacy and underlying mechanisms of ND in wound healing remain unknown. In addition, desirable carriers suitable for the local application of ND remain elusive.

In this study, apart from bactericidal ability, we initially found that ND could modulate fibroblast behaviors by promoting migration and differentiation. To favor local application and controlled release of ND in wound sites, we engineered three kinds of nanodefensin-encased hydrogels (NDEFgels) using FDA-approved hydrogel materials, including triglycerol monostearate (TG18), alginate (Alg) and Pluronic F127 (Plu), and thereafter demonstrated that the Plu hydrogel was the optimal carrier for ND delivery. Then, we verified the therapeutic efficacy of NDEFgel based on Plu hydrogel in full-thickness excisional murine models complicated with bacterial infection. The results showed that NDEFgel significantly accelerated wound healing, including improved collagen deposition, angiogenesis, and re-epithelialization, and relieved inflammation and bacterial burden, which were associated with improved fibroblast behaviors. We found that ND-induced promotion of fibroblast migration and differentiation *in vitro* was regulated by Rac1 through sustained cytoskeletal rearrangement. Overall, the present study provides a novel biomaterial, NDEFgel, which targets both bactericidal and pro-regenerative roles and is a promising candidate for translation into a potential prehospital therapy for wound management.

## Methods

### Synthesis of HD5 and ND

HD5 and ND were synthesized as described previously 20. Briefly, an optimized 2-(1-H-benzotriazol-1-yl)-1,1,3,3-tetramethyluronium hexafluorophosphate (HBTU) activation/diisopropylethylamine (DIEA)* in situ* neutralization protocol was adopted to synthesize peptides on an ABI 433A peptide synthesizer. A correct pattern of disulfide bridges was achieved using autoxidation with crude peptides at 0.25 mg/mL in 50 mM Tris/HCl, 2 M urea, 25% N, N-dimethylformamide, and 3 mM reduced and 0.3 mM oxidized glutathione (pH 8.3) overnight at room temperature. The obtained products were purified by reverse-phase high-performance liquid chromatography (RP-HPLC), and molecular weights were verified by electrospray ionization mass spectrometry (ESI-MS).

### Synthesis of an ND-containing TG18 hydrogel formulation (ND-TG18)

ND-TG18 hydrogel was synthesized as described previously with slight modifications 21. Briefly, 100 mg of TG18 (AK Scientific) was mixed with 1 mL of DMSO-water mixture (1:4 volume ratio), and the resultant mixture was heated to 70 °C until TG18 dissolved. Afterward, the heated TG18 solution in a glass vial was placed at room temperature for 15-30 min to allow hydrophobic assembly, resulting in the formation of hydrogel. To achieve ND functionalization, the blank TG18 hydrogel was heated to obtain an injectable solution, and then the TG18 solution was mixed with ND solution at a high concentration to give a final concentration of 50 μg/mL for ND. The ND-containing TG18 solution was placed at room temperature until hydrogel formation. Finally, the ND-TG18 hydrogel formulation was obtained according to the above protocols.

### Synthesis of an ND-containing Alg hydrogel formulation (ND-Alg)

ND-Alg hydrogel was synthesized as described previously with slight modifications 22. Briefly, 42 mg of sodium alginate (AK Scientific) was mixed with 1 mL of double-distilled water (ddH_2_O) until dissolved. Then, 250 μL of ND solution (200 μg/mL) was well mixed with an equal volume of the Alg solution to produce stock drug-Alg solution. Ionic gelation was triggered by adding 200 μL of saturated calcium sulfate dihydrate (Sigma) solution to the equal volume of drug-Alg solution. Afterward, the mixture was homogenized to ensure homogenous distribution of calcium ions and crosslinking of the Alg chains. Finally, the ND-Alg hydrogel formulation was obtained according to the above protocols.

### Synthesis of an ND-containing Plu hydrogel formulation (ND-Plu)

The PEO-PPO-PEO copolymer Pluronic F-127 (Sigma) was mixed with ddH_2_O to give a final concentration of 25% (w/v). The copolymer suspension was placed overnight at 4 °C to swell, followed by ultrasonic concussion on ice for 30 min. Then, the copolymer suspension was transferred to a -20 °C environment for 5 min to accelerate copolymer dissolution. Afterward, the suspension underwent ultrasonic concussion on ice until a clear and transparent Plu nanogel solution was obtained. The obtained nanogel solution was then mixed with ND solution at a high concentration to give a final concentration of 50 μg/mL for ND in the nanogel solution, thus obtaining the ND-Plu hydrogel formulation. The ND-Plu hydrogel formulation achieved full physical crosslinking at 37 °C and was stored at 4 °C.

### Rheology

Rheological experiments were performed to verify the thermosensitive properties of hydrogels using an MCR302 rheometer (Anton Paar, Austria). 2 mL of cooled samples (stored at 4 ºC) were placed between parallel plates with a diameter of 25 mm and with a gap of 1.0 mm. The temperature was gradually raised from 4 to 50 ºC at a heating rate of 2 ºC/min. The storage modulus G' (elastic response) and loss modulus G” (viscous behavior) were monitored at a strain of 1% with a frequency of 0.1 Hz.

### Dynamic light scattering (DLS)

To reveal the average particle size, the hydrodynamic diameters (d_h_) and number distribution of ND (100 μg/mL) were monitored by the DLS principle using a Malvern Nano-ZS90 instrument (Malvern, UK).

### *In vitro* antimicrobial activity assay

The antimicrobial activity of ND and traditional antibiotics was revealed by OD value variation, as previously described 23. Briefly, a single colony of methicillin-resistant *Staphylococcus aureus* (MRSA) ATCC 43300 in Luria-Bertani (LB) agar plates was cultured in 5 mL of LB at 37 °C and 200 rpm in an incubator. After growing for 6-8 h, the bacteria were washed three times and then adjusted to an approximate OD value of 0.4 at 600 nm using a Molecular Devices SPECTRA MAX 190 microplate reader (Sunnyvale, CA, USA). The adjusted bacterial suspension was incubated with ND, kanamycin, ampicillin, and vehicle at 37 °C and the duration of treatments was 1 h, during which the OD values were dynamically monitored by a Molecular Devices SPECTRA MAX 190 microplate reader (Sunnyvale).

### *In vitro* hydrogel degradation

The *in vitro* degradation of hydrogels was determined by performing a weight remaining (%) experiment, as described by previous literature 24. Briefly, samples of 500 μL of various hydrogel formulations were added into tubes and incubated in a metal bath at 37 °C. After gelation, the original weight of each hydrogel formulation was recorded as W_0_. 1 mL of pre-equilibrated PBS (pH 7.4) was gently laid over the surface of the hydrogels and incubated in a metal bath at 37 °C with continuous gentle shaking. The weight of the remaining hydrogel samples (W_t_) was recorded at regular time intervals (1, 2, 4, 6, 12, 24, 36, and 48 h) after removing the supernatant. The *in vitro* degradation assay was performed in triplicate. The weight remaining (%) was calculated as:


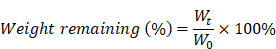


### *In vitro* release of ND from hydrogels

*In vitro* release profiles of the physically entrapped ND from hydrogels were studied by a membrane-less experiment as described previously 24. Briefly, samples of 500 μL of various hydrogel formulations were added into tubes and incubated in a metal bath at 37 °C. After gelation, 500 μL of pre-equilibrated PBS (pH 7.4) was gently laid over the surface of the hydrogels and incubated in a metal bath at 37 °C with continuous gentle shaking. To determine the released ND, supernatant was withdrawn at regular time intervals (1, 2, 4, 6, 12, 24, 36, 48 h) and replaced with pre-equilibrated fresh PBS (50 μL). The concentrations of ND in supernatants at different time points were detected by a Pierce™ BCA Protein Assay Kit (Thermo, USA). The release assay was performed three times.

### Viable count assay

The viable count assay was conducted as described previously 9. Briefly, a single colony of MRSA ATCC 43300 in LB agar plates was cultured in 5 mL of LB at 37 °C and 200 rpm in an incubator. After growing for 6-8 h, the bacteria were washed three times and then adjusted to a bacterial concentration of 2×10^8^ CFU/mL. 500 μL of bacterial suspension (2×10^8^ CFU/ml) was fully mixed with 500 μL of drug-loaded or nondrug-loaded hydrogels and incubated at 37 °C for 2 h. Following incubation, serial 10-fold dilutions were performed, and 5 μL of each dilution was plated on LB agar plates and incubated overnight at 37 °C, followed by detection of CFU.

### Virtual colony-count assay

The virtual colony-count assay was performed as described previously 17, 20, 25. Briefly, a single colony of MRSA ATCC 43300 in LB agar plates was cultured in 5 mL of LB at 37 °C and 200 rpm in an incubator. After growing for 6-8 h, the bacteria were washed three times. To determine the bactericidal activity of ND in the presence of different hydrogel materials, 500 μL of bacterial suspension (2×10^8^ CF U/mL) was fully mixed with 500 μL of drug-loaded or nondrug-loaded hydrogels and incubated at 37 °C for 2 h. 10 μL of the supernatant from each bacteria-gel mixture was then taken and diluted 1000-fold with ddH_2_O. 100 μL of this diluted solution was added to a well in a 96-well plate, and 100 μL 2-fold LB medium was added. OD measurement was obtained in the microplate reader. We then monitored the growth curve of the bacteria in each well at 600 nm overnight at 37 °C and used an algorithm developed previously 25 to calculate the bacterial survival rate of MRSA in each hydrogel. To monitor the antimicrobial kinetics of each ND-loaded hydrogel, 500 μL of bacterial suspension (2×10^8^ CFU/mL) was gently laid over the surface of each ND-loaded hydrogel (500 μL) and incubated in a metal bath at 37 °C with continuous gentle shaking. To measure the bactericidal ability of released ND at different time points, the supernatant was withdrawn at regular time intervals (1, 2, 4, 6, 12, 24, 36, and 48 h) and replaced with pre-equilibrated fresh PBS (50 μL). 50 μL of supernatant samples was then added to a 96-well plate and supplemented with 50 μL of PBS and 100 μL of 2-fold LB medium. OD measurements were taken in a microplate reader at 600 nm overnight. Bacterial survival rates were calculated by the aforementioned method.

### Cell culture

The BALB/c clone 3T3 A31 cell line (mouse embryo fibroblast cell line) was purchased from the Type Culture Collection of the Chinese Academy of Sciences, Shanghai, China. Cells were cultured in standard medium comprising Dulbecco's modified Eagle's medium (DMEM, C11995500BT; Gibco) supplemented with 10% fetal calf serum (FBS, 10099-141; Gibco) with 1% penicillin/streptomycin (P/S) (15140-122; Gibco) in an incubator at 37 °C with 5% CO_2_.

### *In vitro* scratch assay

Fibroblast 3T3 cells were cultured overnight on 12-well plates at a cell density of 4×10^5^ cells/well. The cell monolayers were scraped in a straight line using a 200-µL pipette tip. To evaluate the ability of HD5 and ND to induce cell migration, cells were stimulated with HD5 (final concentration of 12.5 μg/mL), ND (final concentration of 12.5 μg/mL), and an equal volume of PBS as a control. To verify the role of Rac1 in HD5/ND-induced migration, cells were treated with a 50 μM concentration of the Rac1-selective inhibitor NSC23766 for 30 min before stimulation with HD5/ND (final concentration of 12.5 μg/mL). The cell plates were observed with an Olympus IX53 inverted microscope (Olympus, Tokyo, Japan) using a 4× objective at 0 and 24 h. The unclosed area was quantified at the indicated time using ImageJ software. The unclosed area ratio was calculated as the ratio of the unclosed area (t = 24 h) to the wounded area (t = 0 h).

### Immunofluorescence

Cytoskeletal morphology was characterized by fluorescent staining of filamentous actin (F-actin). In brief, fibroblast 3T3 cells were cultured overnight on confocal dishes at a cell density of 1×10^5^ cells/well. Cells were stimulated with HD5 (12.5 μg/mL) or ND (12.5 μg/mL) with/without pretreatment with NSC23766 (50 μM) and an equal volume of PBS as a control for 3 h. After being washed with PBS three times, the cells were fixed with 4% paraformaldehyde for 10 min, followed by permeabilization with 0.5% Triton X-100 for 5 min. Then, the cell samples were stained with rhodamine-conjugated phalloidin (Life Technologies) to visualize the F-actin arrangement, which was observed using an Olympus FV1000 inverted confocal microscope (Olympus, Tokyo, Japan).

The transformation of fibroblasts into myofibroblasts was characterized by immunofluorescence staining of α-smooth muscle actin (α-SMA). In brief, fibroblast 3T3 cells were cultured overnight on confocal dishes at a cell density of 2×10^5^ cells/well. Cells were stimulated with HD5 (12.5 μg/mL) or ND (12.5 μg/mL) with/without pretreatment with NSC23766 (50 μM) and an equal volume of PBS as a control for 3 h. After being washed with PBS three times, the cells were fixed with 4% paraformaldehyde for 10 min, followed by permeabilization with 0.5% Triton X-100 for 20 min. Thereafter, the cell samples were blocked with 10% normal goat serum for 1 h at room temperature, followed by staining with rabbit anti-α-SMA (1:200 (CST)) overnight at 4 °C. A fluorescent signal was generated by labeling with a FITC-conjugated secondary antibody. Finally, the expression and arrangement of α-SMA were visualized using an Olympus FV1000 inverted confocal microscope (Olympus, Tokyo, Japan).

### Murine wound healing model

Seven-week-old BALB/c mice were purchased from Shanghai SLAC Laboratory Animal Co., Ltd. All animal experiments were conducted under the approval of the Animal Advisory Committee at Zhejiang University. A full-thickness excisional murine wound model was used to study wound healing 23, 26-28. The mice were individually raised in cages in a standardized environment with proper animal treatment and then randomly divided into four groups: (1) the non-treatment group, (2) the Prontosan treatment group, (3) the NDEFgel treatment group, and (4) the sham-operated group. Hair was removed from the backside of mice using a depilatory cream with anaesthetization through an intraperitoneal injection of ketamine (100 μg/kg)/xylazine (20 μg/kg) at least 1 day prior to surgery. The mice in the sham-operated group did not undergo cutaneous wound surgery, while the other groups underwent cutaneous wound surgery under anesthesia (ketamine (100 μg/kg)/xylazine (20 μg/kg)). A full-thickness rectangular wound (10 mm×10 mm) was constructed using a surgical scalpel for each mouse on the backside, followed by a challenge with 1×10^7^ CFU of MRSA ATCC 43300 to cover the injury area uniformly. After 1 h of persistent bacterial infection, nothing was added to the wound bed for the non-treatment group, the Prontosan treatment group was treated with 100 μL of Prontosan, and the NDEFgel treatment group was treated with 100 μL of NDEFgel (ND concentration, 50 μg/mL). Then the therapeutics were fixed with a transparent dressing (SIMP, Shanghai, China). The therapeutics were changed every 2 days. Wounds were briefly cleaned with saline when changing therapeutics. After 4, 10, and 16 days, the wound areas were observed and photographed. All mice in the four groups were sacrificed after 16 days of treatment. Skin tissue samples were excised and used for bacterial burden measurement, histopathological analysis, Masson trichrome staining, Picrosirius Red staining, immunohistochemical analysis, ultrastructural characterization, RNA isolation, and protein analysis.

To compare the therapeutic efficacy of NDEFgel with blank gel and ND solution, murine wound models complicated with MRSA infection were established according to the above protocols and received non-treatment, blank gel treatment, ND solution treatment, or NDEFgel treatment. Then, the therapeutics were fixed with a transparent dressing (SIMP, Shanghai, China). The therapeutics were changed every 2 days. Wounds were briefly cleaned with saline when changing therapeutics. After 4, 10, and 16 days, the wound areas were observed and photographed.

### Bacterial burden measurement

Skin tissue samples collected 16 days post-wounding were homogenized using an IKA T10 basic homogenizer (IKA, Germany) and diluted in sterile PBS. The homogenate or its dilutions were then inoculated on LB plates. The number of CFUs was counted after overnight incubation at 37 °C, and the results were expressed as CFU per gram of tissue.

### Histopathological analysis

Paraffin-embedded microtome-cut sections (4-μm-thick) were stained with hematoxylin and eosin (H&E) and Masson's trichrome staining reagents to reflect the regenerative status of cutaneous tissues under observation with an Olympus VS120 microscope (Olympus, Tokyo, Japan) at a magnification of 200×. Semiquantitative skin regenerative scores were based on the five aspects described previously, with proper modification 29. Briefly, skin regeneration was assigned scores separately for epidermal homeostasis (0, severe hyperplasia; 1, moderate hyperplasia; 2, mild hyperplasia; and 3, normal), neovascularization (0, almost absent; 1, occasional presence; 2, light scattering; and 3, abundant), collagen deposition (0, 0-10% positive area; 1, 10-20% positive area; 2, 20-30% positive area; 3, 30-40% positive area; and 4, > 40% positive area), granulation proliferation (0, almost absent; 1, mild; 2, moderate; and 3, abundant), and inflammatory infiltration (0, severe; 1, moderate; 2, mild; and 3, almost absent). The positive area of collagen deposition was calculated by ImageJ software in the local field. Two examiners performed histopathological analysis without prior knowledge of the experimental interventions.

To characterize the composition of collagen, paraffin sections of cutaneous tissues were stained with Picrosirius Red. The obtained Picrosirius-Red stained sections were observed and photographed by a Polarizing Optical Microscope with Heating Stage (Nikon, Japan). The proportion of different types of collagen was calculated using ImagePro Plus version 4.5 based on photographs of Picrosirius-Red staining sections.

To evaluate organ injury, the liver, spleen, lung, and kidney were separated to prepare paraffin-embedded sections (4 μm thick) with H&E staining. Histopathology images were acquired with an Olympus BX61 upright microscope (Olympus, Tokyo, Japan) using a 20× objective.

### Immunohistochemical analysis

Paraffin sections of cutaneous tissues were employed for immunohistochemical examination. The sections were autoclaved in 0.01 M citrate buffer (pH 6.0) for 10 min and then immersed in 1% bovine serum albumin (Sigma, USA) for 30 min 30. Subsequently, they were incubated overnight at 4 °C in a 1:2000 dilution of anti-CD31 antibody (Huabio, China); a 1:200 dilution of anti-F4/80 antibody (Huabio, China); a 1:200 dilution of anti-Ly6G antibody (Huabio, China); a 1:2000 dilution of anti-cytokeratin 14 (K14) antibody (Abcam, UK); a 1:50 dilution of anti-RAC1/2/3 antibody (Huabio, China); and a 1:50 dilution of anti-α-SMA antibody (Huabio, China). An EnVision Kit (DAKO Japan Inc., Japan) was used for visualization. The resultant immunohistochemical sections were observed and photographed by an Olympus VS120 microscope (Olympus, Tokyo, Japan) at a magnification of 200×. The positive area for each marker was calculated using ImageJ IHC Profiler.

### Scanning electron microscopy (SEM)

To visualize the 3D network structure, NDEFgel and blank gel were characterized using a Nova Nano 450 field-emission SEM (Thermo FEI, USA) after freezing-drying and sputtering the gold layer.

To visualize the ultrastructure of the cornified layer and collagen array, fresh skin tissues were excised and mixed with 2.5% glutaraldehyde at room temperature for 2 h, followed by overnight incubation at 4 °C. The skin tissues were washed with PBS three times, fixed with 1% OsO_4_ for 1.5 h, and post-dehydrated through a graded ethanol series. The samples were dried with a LEICA EM CPD300 critical point dryer (LEICA, Germany) before coating with platinum. Finally, the skin samples were characterized using a Nova Nano 450 field-emission SEM (Thermo FEI, USA).

### Transmission electron microscopy (TEM)

To characterize the supramolecular nanostructure of ND, ND solution with a concentration of 100 μg/mL was dropped onto a 300-mesh copper grid coated with carbon. After 5 min of adsorption, the residual solvent was removed using filter papers. The copper grid was dried at room temperature and then underwent negative staining through a 2 wt. % aqueous uranyl acetate solution. After that, the ultrastructure of ND was observed under a Tecnai G2 Spirit 120 kV transmission electron microscope (Thermo FEI, USA).

To characterize the ultrastructure and distribution of hemidesmosomes attached to the basement membrane, fresh skin tissues were excised and mixed with 2.5% glutaraldehyde at room temperature for 2 h followed overnight at 4 °C. After washing with PBS three times, the skin samples were incubated with 1% OsO_4_ for 1 h, dehydrated using a graded ethanol series, and embedded in epoxy resin. The skin tissue samples were sliced using an ultramicrotome (Leica EM UC7). Ultrathin sections (100 nm) were mounted on copper grids and contrasted with 4% uranyl acetate and lead citrate. The copper grids were observed with a Tecnai G2 Spirit 120 kV TEM (Thermo FEI, USA).

### Western blotting

Tissue or cell samples were lysed in RIPA lysis buffer (Applygen, Beijing, China) containing phenylmethylsulfonyl fluoride (PMSF, Beyotime, Shanghai, China). Equal amounts of protein, which were determined by a BCA Protein Assay Kit (Thermo Pierce, Waltham, USA), were separated via 12% Bis-Tris NUPAGE gradient gels with MOPS running buffer and then transferred onto polyvinylidene difluoride (PVDF) membranes (Thermo Fisher, Waltham, USA). After blocking with 5% skim milk in Tris-buffered saline (TBS) for 1 h, the membranes were blotted with primary antibodies on a shaker overnight at 4 °C. Primary antibodies were as follows: mouse anti-Rac1 (1:1000, Thermo), rabbit anti-α-SMA (1:1000, CST), rabbit anti-EGFR (1:1000, Abcam), rabbit anti-EGFR (phosphor Y1068) (1:1000, Abcam), and rabbit anti-GAPDH (1:1000, CST). The membranes were washed three times using TBS with 0.05% Tween-20 for 10 min each, followed by incubation with corresponding secondary horseradish peroxidase (HRP)-conjugated antibodies (1:5000, Lianke Technology) for 1 h at room temperature. Blots were developed using an enhanced chemiluminescence kit (Biological Industries) and visualized via X-ray films. The levels of protein expression were quantified by ImageJ software.

### Quantitative reverse transcription-polymerase chain reaction (qRT-PCR)

Total RNA was isolated from skin tissues using TRIzol reagent (Life Technologies). RNA was reverse transcribed into cDNA using a reverse transcription kit (Takara, Kyoto, Japan). The obtained cDNA was subjected to DNA amplification using the Roche 480 PCR System (Roche, USA), in which TB GreenTM premix Ex TaqTM II (Takara) was employed to quantify PCR amplification products. The primer sets used for amplification are shown in Supplementary Table 1. The relative expression of target genes was calculated by the 2^-ΔΔCT^ model. β-actin was used as a reference gene.

### Statistical analyses

The results are expressed as the mean ± SD. A two-tailed Student's *t*-test, a paired *t*-test, or one-way analysis of variance with Bonferroni corrections was performed to characterize statistical significance. Statistical analyses were performed using GraphPad Prism 7.0 software (GraphPad Software Inc., San Diego, USA). Principal component analysis (PCA) was conducted and visualized using Biovinci version 1.1.1 (BioTutoring Inc., San Diego, CA, USA). P values < 0.05 were considered statistically significant.

## Results

### ND synthesis and* in vitro* characterization

**Figure 1:**
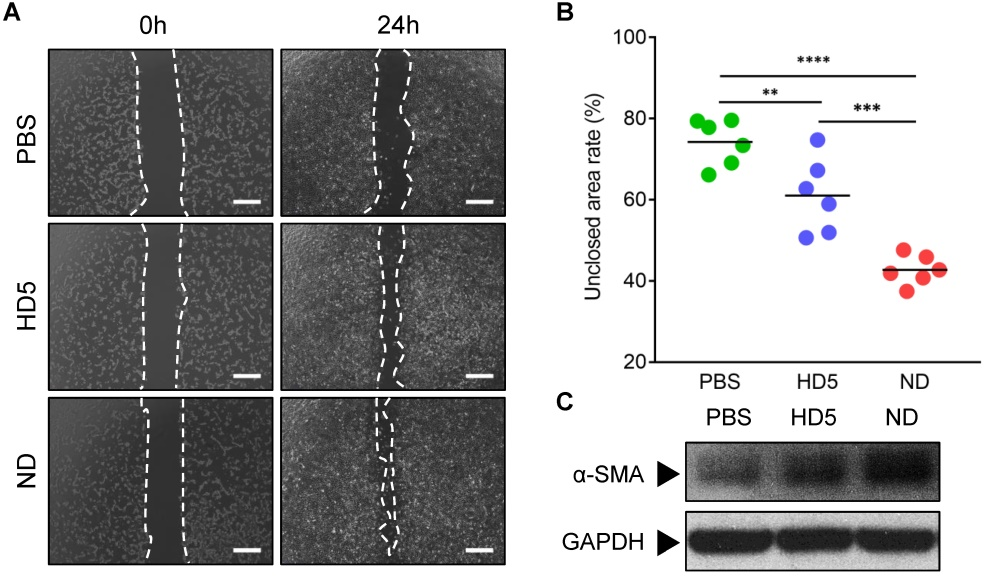
**ND promotes fibroblast migration and favors differentiation into myofibroblasts.** (A) Representative images of fibroblast 3T3 cells migration upon the incorporation of PBS, HD5 (12.5 µg/mL), and ND (12.5 µg/mL), determined using an *in vitro* scratch wound assay. Scale bars, 1 mm. (B) Quantitative analysis of the unclosed scratch wound area. n = 6 per group. (C) Western blotting detection of α-SMA expression levels in fibroblast 3T3 cells after stimulation with PBS, HD5 (12.5 µg/mL), or ND (12.5 µg/mL). GAPDH was adopted as an internal reference. Data are shown as the mean ± SD. Statistical analyses were calculated by one-way analysis of variance with the Bonferroni correction for multiple comparisons. *P < 0.05; **P < 0.01; ***P < 0.001; ns, not significant.

Natural HD5 and ND were successfully synthesized via solid-phase peptide synthesis (SPPS) in this study, as validated by the results of ESI-MS and RP-HPLC which indicated that both synthesized peptides were of the expected molecular weight (**Figure S1-S2**), with a purity of > 98% (**Figure S3-S4**). Our synthesized ND used in this study that, in the aqueous phase, is capable of self-assembling into a supramolecular nanostructure, termed ND (**Figure S5A**), with a hydrodynamic diameter (d_h_) ranging from 70-200 nm (**Figure S5B-C**), which is consistent with our previous reports 20. The above physicochemical characterizations verified the correct synthesis of HD5 and ND which were accepted for subsequent investigations. Our previous study proved that ND exerts significantly more vigorous bactericidal activity than natural HD5 20. In this study, we demonstrated that ND exhibited stronger *in vitro* bactericidal activity against MRSA than kanamycin and ampicillin (**Figure S5D**). However, their activity in wound healing is unknown. To evaluate this potential, we performed a wound scratch assay to determine whether it is possible to promote the migration of the fibroblast 3T3. We found that ND significantly induced fibroblast 3T3 cell migration upon 24-h incorporation of ND, and its contribution exceeded the effect of HD5 (**Figure 1A-B**). In addition, *in vitro* stimulation with ND contributed to elevated expression of α-SMA, indicating the transformation of fibroblasts into myofibroblasts (α-SMA-positive fibroblasts) (**Figure 1C and Figure S6**). The effect of ND was significantly higher than that of HD5 (**Figure 1C and Figure S6**). Collectively, the above results demonstrated that ND exhibited dual functions targeting both bacterial elimination and fibroblast behaviors.

### Formulation of NDEFgel and physical properties

**Figure 2:**
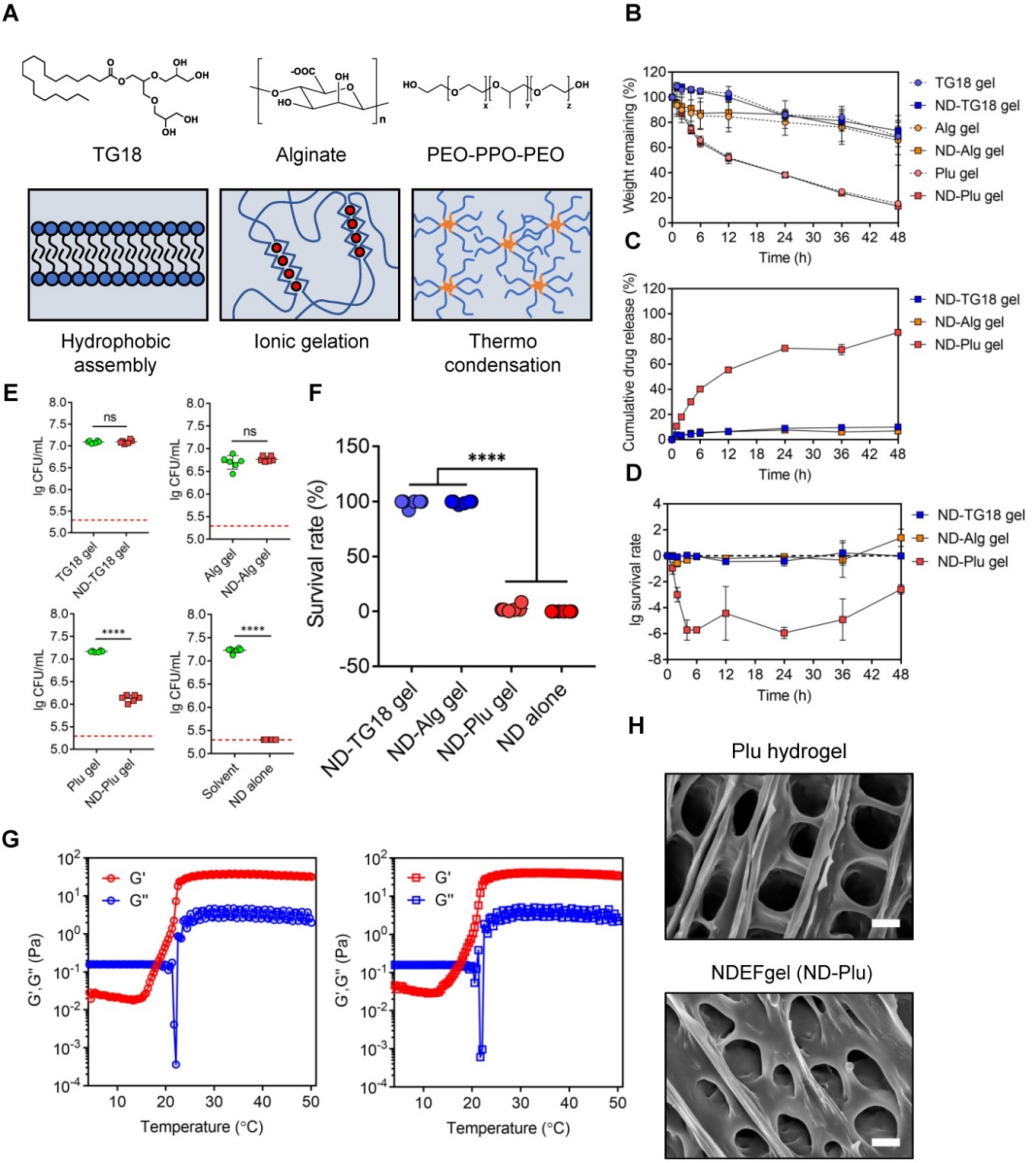
** Screening of hydrogel platforms for ND delivery.** (A) Multifaced hydrogel platforms based on different assembling mechanisms including hydrophobic assembly (TG18), ionic gelation (alginate), and thermocondensation (PEO-PPO-PEO). (B) Degradation kinetics of blank or ND-contained hydrogel formulations incubated at 37 °C measured by weight remaining (%). Values are presented as mean ± SD, n = 3. (C) *In vitro* drug release profiles of ND-loaded hydrogels within 48 h, n = 3 per group per time point. (D) *In vitro* antimicrobial kinetics of ND-loaded hydrogels within 48 h, n = 3 per group per time point. (E) Viable count assay detected the live bacteria (MRSA) after fully mixing with various hydrogel formulations (n = 6); nanodefensin aqueous solution, ND alone; nanodefensin-containing Pluronic F127 hydrogel, ND-Plu gel; nanodefensin-containing alginate hydrogel, ND-Alg gel; nanodefensin-containing TG18 hydrogel, ND-TG18 gel. (F) Virtual colony-count assay reported the survival rates of MRSA after fully mixing with various hydrogel formulations (n = 6). (G) Temperature-dependent rheological characterization verified the mechanical properties of the blank hydrogel (left) and NDEFgel (right). (H) SEM reveals the 3D network structure of blank Plu hydrogel and NDEFgel (ND-Plu). Scale bars, 2 µm. Data are shown as the mean ± SD. Statistical analyses were calculated by two-tailed Student's *t*-test and one-way analysis of variance with the Bonferroni correction for multiple comparisons. *P < 0.05; **P < 0.01; ***P < 0.001; ns, not significant.

To adapt to clinical applications, we next intended to select suitable ND hydrogel formulations among FDA-approved hydrogels based on generally recognized as safe (GRAS) compounds 21, 31. We synthesized three kinds of NDEFgels using three GRAS hydrogel materials (**Figure 2A**): 1) TG18, a small molecular amphiphile, that can self-assemble into lamellar hydrogels by hydrophobic forces 21; 2) Alg hydrogel, which is formed by calcium ion-mediated ionic crosslinking (**Figure S7**) 22, 32; and 3) Plu gel, which undergoes a transformation between liquid and gel states in response to temperature changes 33. We initially compared the degradation kinetics of TG18 gel, Alg gel, and Plu gel, together with them when loaded with ND, for 48 h. The results demonstrated that incorporating ND into the three hydrogels did not significantly alter their inherent degradation behaviors (**Figure 2B**). Among these hydrogel formulations, the degradation rates of TG18 gel, Alg gel, and their ND-encased formulations (termed ND-TG18 gel and ND-Alg gel) were significantly slower than those of the Plu gel and ND-Plu gel, and they only degraded by ~20% within 48 h (**Figure 2B**). In contrast, Plu-based gels exhibited distinct time-dependent degradation with ~50% erosion at 12 h and ~80% erosion at 48 h (**Figure 2B**), implying a more favorable drug-release profile compared with TG18 and Alg as drug carriers. As expected, Plu gel indeed achieved ~80% ND release within 48 h, whereas the TG18 gel and Alg gel only released ~10% ND (**Figure 2C**), which is in good agreement with the degradation behaviors. Furthermore, we also monitored the *in vitro* antimicrobial kinetics of each ND-loaded formulation when the MRSA suspension was gently placed onto the hydrogel surface (**Figure 2D**). ND-Plu gel exhibited a rapid time-dependent increase in bactericidal efficacy and then maintained high-performance activity for the whole 48 h (**Figure 2D**), which is very important for wound care to constrain bacterial infection effectively and rapidly. However, both the ND-TG18 gel and ND-Alg gel barely showed differential bactericidal effects within 48 h (**Figure 2D**), despite ~10% of ND being released (**Figure 2C**). Thus, we reasoned that hydrogel materials might disturb the activity of ND. To evaluate this hypothesis, we fully mixed the MRSA suspension with each hydrogel formulation, and then quantified the antimicrobial ability of ND in the presence of different hydrogel materials. The viable count results demonstrated that ND retained its major antimicrobial activity against MRSA in the presence of Plu gel, compared to ND in aqueous solution (**Figure 2E and Figure S8A**). By contrast, ND significantly lost its bactericidal ability in the presence of TG18 and Alg, as indicated by lack of a significant difference in viable bacteria between blank gels and ND-loaded gels (**Figure 2E and Figure S8A**). To support this finding, we also conducted a virtual colony-count assay to calculate the survival rates of MRSA according to the growth curves of MRSA after fully mixing with various ND formulations for 2 h. The results consistently indicated that ND, delivered by Plu gel, still possessed comparable activity to its inherent activity (~97% killing rate for ND-Plu gel *vs* 100% killing rate for ND alone) (**Figure 2F and Figure S8B**). However, formulations using TG18 and Alg indeed dramatically inactivated ND (**Figure 2F and Figure S8B**). Given the above analysis, we selected the Plu hydrogel to formulate ND, and the resultant ND-Plu gel was directly termed NDEFgel in subsequent investigations (**Figure S9**).

To study the influence of ND on the mechanical properties of Plu hydrogel, we monitored the rheological alterations of blank Plu hydrogel and NDEFgel when temperature increased. As shown in **Figure 2G**, both blank Plu gel and NDEFgel showed distinct and similar temperature-responsive alterations in G' and G”. The G” of blank Plu gel increased with increasing temperature ranging from 21.3 °C to 30.5 °C, while the incorporation of ND did not affect the thermosensitive features of G”, which increased when the temperature was enhanced from 20.9 °C to 30.1 °C. When the temperature was raised from 15.2 °C to 27.1 °C, the G' of blank Plu gel continuously increased. However, G' of NDEFgel was thermosensitive in the temperature range of 12.9 °C to 27.8 °C, implying that the incorporation of ND might slightly broaden the thermosensitive range of G'. Of further note, both G' and G” of blank Plu gel and NDEFgel had already reached homeostasis at 37 °C, and G' > G” suggested gel formation. In parallel, SEM showed that NDEFgel could assemble* in situ* into a three-dimensional (3D) network structure at 37 °C, similar to the blank Plu hydrogel (**Figure 2H**). The above physical characterizations suggested its use as an injectable nanogel formulation and/or *in situ* scaffold for local therapeutics.

### Efficient* in vivo* bacterial control and wound healing by NDEFgel

**Figure 3:**
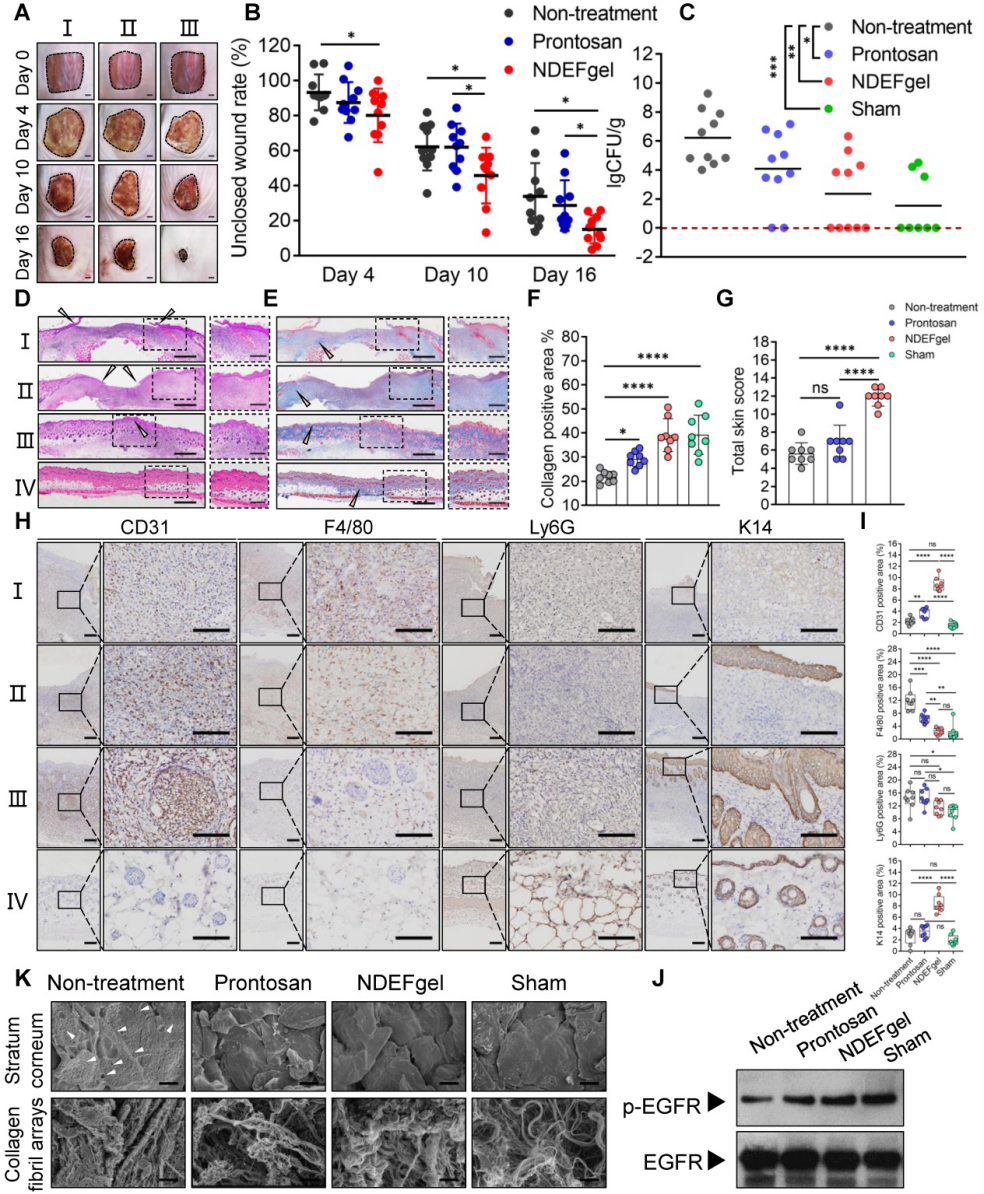
** NDEFgel successfully accelerates wound recuperation in murine full-thickness wound models complicated with MRSA infection.** (A) Macroscopic imaging of wounds immediately following surgery (Day 0) and after 4, 10, or 16 days of treatment. I, non-treatment group; II, Prontosan treatment group; III, NDEFgel treatment group. Scale bars, 2 mm. (B) Quantification of unclosed wound rates in the murine wound models after 16 days of treatment. n = 10 per group. (C) Bacterial burden measurements of regenerated skin tissues from murine wound models after treatment for 16 days (n = 10) or sham-operated models (n = 8). Dashed lines indicate the limit of detection. Representative images of haematoxylin and eosin (H&E) staining (D) and Masson's trichrome staining (E) of skin tissue sections from murine wound models without treatment (I), with Prontosan (II) or NDEFgel treatment (III), and from sham-operated models (IV). Gray arrowheads mark the wound bed, yellow arrowheads mark collagen fibrils. Scale bars, 400 µm. Dashed boxes indicate local fields (LF). Scale bars, 200 µm. (F) Quantification of the positive area of collagen deposition according to Masson's trichrome staining images (n = 8). (G) Total histopathological scores of skin tissues from murine wound models according to results of H&E staining and Masson's trichrome staining. n = 8 per group. (H) Immunohistochemical analysis of cutaneous tissues from non-treatment (I), prontosan-treated (II), NDEFgel-treated wound beds (III) or sham-operative skins (IV) to present the expression and distribution of vascular endothelial cells (CD31^+^), macrophages (F4/80^+^), neutrophils (Ly6G^+^), and keratin (K14^+^). Scale bars, 100 µm. Blank line boxes highlight LF. Scale bars, 50 µm. (I) Quantitative analysis of immunohistochemical sections, conducted using ImageJ IHC Profiler. n = 8 per group. (J) Western blotting was used to detect the protein levels of p-EGFR and EGFR in skin tissue from the non-treatment, Prontosan treatment, NDEFgel treatment, or sham-operated groups. (K) SEM revealed the ultrastructure of cornified layers and collagen fibril arrays of skin tissues from the non-treatment, Prontosan treatment, NDEFgel treatment, or sham-operated groups. White arrowheads, “alveolate” holes. Scale bars, 10 µm. Data are shown as the mean ± SD. Statistical significance was analyzed by a two-tailed Student's *t*-test or one-way analysis of variance with the Bonferroni correction for multiple comparisons. *P < 0.05; **P < 0.01; ***P < 0.001; ns, not significant.

To evaluate the clinical potential, we compared the* in vivo* therapeutic efficacy between NDEFgel and the clinical hydrogel dressing Prontosan in full-thickness excisional murine wound models with MRSA infection. Our results showed that 4 days after wound treatment, NDEFgel treatment gradually outperformed expectations (**Figure 3A**), resulting in ~23% wound closure, which dramatically outpaced that the results of the non-treatment (~7% closure rate), and Prontosan treatment groups (~13% closure rate) (**Figure 3B**). After 10 days of treatment, NDEFgel treatment contributed to a ~55% wound closure rate, which was significantly higher than that of the non-treatment control (~39% closure rate). Mice that received Prontosan hydrogels (~33% closure rate) showed no significant difference from the non-treatment control. NDEFgel therapy maintained its inherent superiority, with a wound closure rate of ~83% at day 16, while the non-treatment and Prontosan groups exhibited closure rates of ~62% and ~66%, respectively. In contrast, application of blank gel did not significantly accelerate wound healing relative to untreated mice, while treatment using ND solution provided relatively more accelerated wound closure than that of blank gel and untreated control (**Figure S10A-B**). Nevertheless, NDEFgel exhibited better therapeutic efficacy than ND solution, suggesting the importance of rational hydrogel delivery (**Figure S10A-B**). Of further note, determination of cutaneous bacterial burden demonstrated that although both NDEFgel and Prontosan could attenuate the cutaneous bacterial burden in comparison to the non-treatment control (**Figure 3C**), the efficiency of the NDEFgel treatment was significantly superior, as indicated by the lowest bacterial level showing no significant difference from the sham-operated controls. Although the average cutaneous bacterial burden in blank gel- and ND solution-treated mice shows slightly reduction in comparison to that of untreated mice, there was no significantly statistical difference (**Figure S10C**).

H&E staining of skin tissue sections obtained from murine wound models for NDEFgel- and Prontosan-treated conditions and untreated and sham-operated control groups are shown in **Figure 3D**. NDEFgel treatment significantly decreased the length of the wound bed, with the appearance of various cutaneous appendages similar to those of healthy skin in the sham-operated control. Masson's trichrome staining analysis clearly showed that the application of NDEFgel remarkably accumulated abundant collagen within the wound beds (**Figure 3E-F**). Semiquantitative histological scoring based on histopathological sections showed the ameliorative effects of the regenerative process with NDEFgel treatment relative to all other groups (**Figure S11A**), especially in terms of collagen deposition. Furthermore, PCA of the histopathological scoring illustrated a relatively distinct separation between the NDEFgel and the non-treatment/Prontosan datasets, suggesting a significant histopathological difference in regenerative processes between the NDEFgel and other groups (**Figure S11B**), which also conformed to the results of the total skin score (**Figure 3G**). Although the application of Prontosan enhanced collagen deposition, both the PCA and total skin score did not support any advantages in terms of accelerating wound healing. In addition, vascular endothelial cells (CD31-positive cells) were much more abundant in NDEFgel-treated skin than in other groups (**Figure 3H-I**), suggesting stronger angiogenesis. Infiltration of macrophages (F4/80-positive cells) was significantly decreased by NDEFgel treatment (**Figure 3H-I**), which demonstrated attenuated inflammation. Although Prontosan could also diminish macrophage infiltration, the effect was significantly weaker than that of NDEFgel. Nevertheless, neutrophil (Ly6G-positive cell) infiltration did not show a significant difference between each intervention group (**Figure 3H-I**). In addition, NDEFgel exhibited superior re-epithelialization in comparison to non-treatment and Prontosan treatment, as indicated by the high expression of cytokeratin 14 (K14) (**Figure 3H-I**). These results demonstrated the excellent wound-healing performance of NDEFgel.

### Improved EGFR signaling and ultrastructural recovery

The epidermal growth factor receptor (EGFR) pathway plays pivotal physiological roles in cell growth, proliferation, and differentiation, and its activation relies on its phosphorylation 34. Consequently, the level of phosphorylated EGFR (p-EGFR) can be regarded as a marker that characterizes the tissue growth ability in regenerative medicine. We monitored the p-EGFR level of regenerated skin tissues among groups using western blotting. The data revealed that higher levels of p-EGFR were exhibited in NDEFgel-treated wounds than in wounds treated with other treatments (**Figure 3J and Figure S12**).

Structurally, full-thickness skin consists of epidermis and dermis, as well as a subcutaneous layer (**Figure S13**). The epidermis can be divided into three parts: the cornified region, the spinous, and basal layers 35; the dermis contains abundant collagen fibrils that contribute to the biomechanical properties of skin 36, 37. Under observation of SEM, cutaneous ultrastructure showed a smooth cornified layer with a regular morphology, similar to that of the sham-operated group, in both the Prontosan and NDEFgel-treated groups (**Figure 3K**), while the non-treatment group showed a rough cornified layer with numerous “alveolate” holes (**Figure 3K**, white arrowheads). Similar to sham-operated animals, collagen fibrils with fluffy, dense, and regular arrays were more abundant in the regenerated skin of NDEFgel-treated mice than in that of the Prontosan-treated and non-treatment mice. To reveal cutaneous fragility 38, we employed TEM to observe hemidesmosomes attached to the basement membrane that supports the tight junctions between the epidermis and dermis (**Figure S13**). As shown in **Figure S14**, NDEFgel therapy resulted in more hemidesmosomes attached to the basement membrane, whose morphology was closer to the regular array of hemidesmosomes in undamaged skin than that of Prontosan-treated and non-treatment groups. Taken together, our results showed that treatment with NDEFgel resulted in the regeneration of more resilient skin tissue than that induced by Prontosan treatment through the activation of the EGFR signaling pathway, improved collagen deposition and arrays, and ultimately influenced the homeostasis of hemidesmosomes on the basement membrane.

### Favorable systemic safety profiles of NDEFgel in wound healing

**Figure 4:**
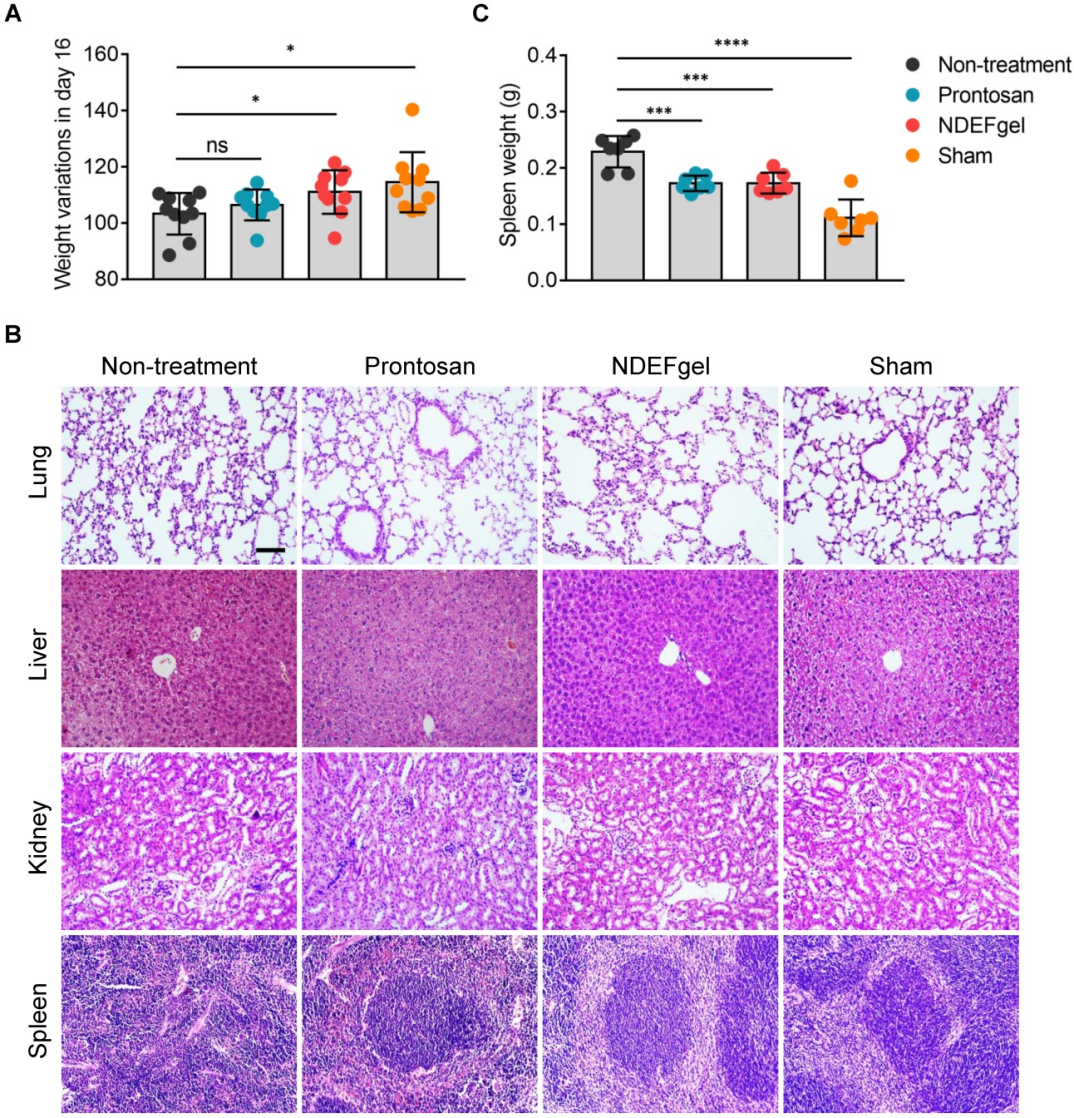
** Favorable systemic safety profiles of NDEFgel in wound healing.** (A) Variation in body weight of the BALB/c mice from non-treatment, Prontosan, or NDEFgel treatment, and Sham-operated groups in day 16 compared with day 0 (n = 10). (B) Representative images of H&E staining sections of lung, liver, kidney, and spleen. Scare bars, 100 µm. (C) Quantification of spleen weight of various groups (n = 7). Data are shown as the mean ± SD. Statistical significance was analyzed by a two-tailed Student's *t*-test or one-way analysis of variance with the Bonferroni correction for multiple comparisons. *P < 0.05; **P < 0.01; ***P < 0.001; ns, not significant.

To understand systemic responses caused by full-thickness excisional wounds with/without therapeutics and evaluate the systemic safety profiles of NDEFgel, we first detected body weight variations in mice after 16 days of treatment. The results showed that both the NDEFgel and sham-operated groups displayed significantly higher increases in body weight than the Prontosan and non-treatment groups (**Figure 4A**), indicating that NDEFgel-treated mice presented more favorable systemic recovery. We further investigated various organs of mice to determine whether any significant pathological changes occurred during treatments. We analyzed the histopathological sections of the liver, lung, kidney, and spleen isolated from treated animals. The results demonstrated that the liver, lung, and kidney did not display significant pathological changes among the groups (**Figure 4B**). Intriguingly, severe pathological changes were observed in the spleens of the non-treatment group, with a dramatic enhancement of spleen weight and disrupted germinal centers (**Figure 4B-C**). In contrast, both the NDEFgel- and Prontosan-treated groups maintained the histological structure of the spleen, with complete germinal centers. Based on the above results and discussion, we revealed that no significant systemic tissue damage existed in NDEFgel-treated mice, which might be attributed to the favorable therapeutic efficacy of NDEFgel on acute wound injury and its high safety profile.

### Development of a pro-regenerative microenvironment by NDEFgel through modulation of fibroblast behaviors

**Figure 5:**
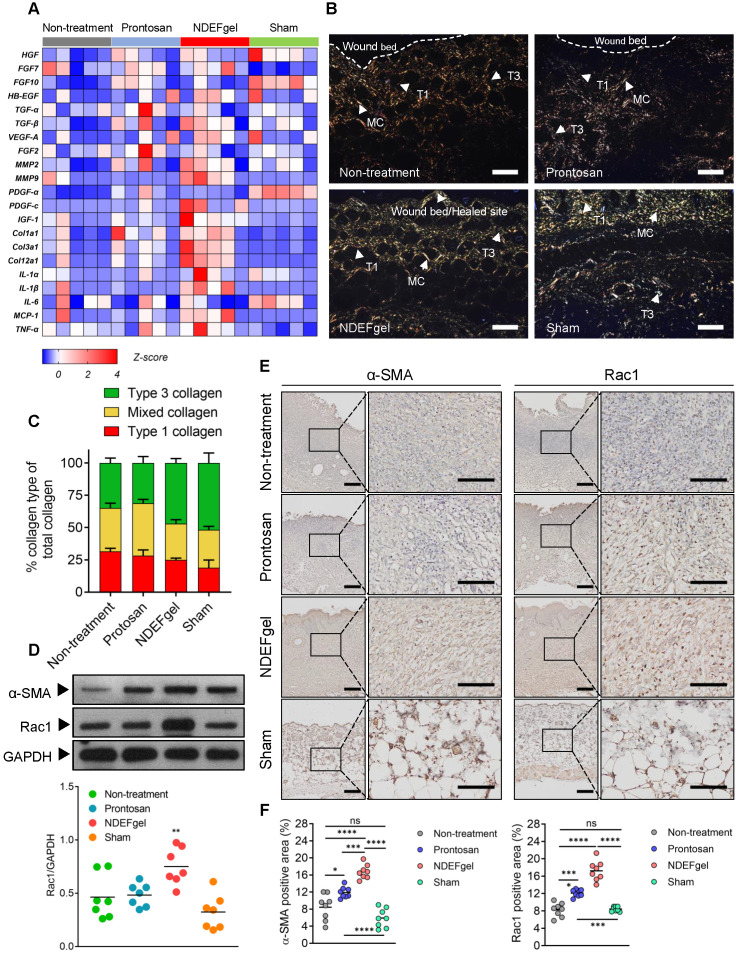
** The pro-regenerative microenvironment developed by NDEFgel treatment was associated with improved fibroblast behaviors.** (A) Transcriptome of skin regeneration-related molecules involved in re-epithelialization, neovascularization, fibroblast and collagen deposition, and the immune response around the wound microenvironment. Full-thickness skin tissues were extracted from wound models without treatment or with Prontosan or NDEFgel treatment and from sham-operated models, as determined by qRT-PCR. Relative mRNA levels were calculated as the quantification relative to sham-operated models according to the 2^-ΔΔCT^ mathematical model. The final data were standardized by Z-score normalization and displayed as Z-scores in the microarray heatmap. n = 5 per group. (B) Representative photomicrographs of Picrosirius Red-stained sections from murine wound skins, observed under a polarization microscope. T1, type I collagen; T3, type III collagen; MC, mixed collagen. Scale bars, 400 µm. (C) Quantification of collagen fiber types at wound beds relative to total collagen, calculated by ImagePro Plus version 4.5 according to results of Picrosirius Red-stained analysis. n = 8 per group. (D) Western blotting detecting the protein levels of α-SMA and Rac1 in skin tissue from the non-treatment, Prontosan treatment, NDEFgel treatment, or sham-operated groups. GAPDH was adopted as an internal reference. n = 7 per group. (E) Immunohistochemical analysis of cutaneous tissues from non-treatment (I), prontosan-treated (II), NDEFgel-treated wound beds (III) or sham-operative skins (IV) to present the expression and distribution of α-SMA and Rac1. Scale bars, 100 µm. Blank line boxes highlight LF. Scale bars, 50 µm. (F) Quantitative analysis of the expression α-SMA and Rac1 in wound beds or sham-operative skins according to immunohistochemical sections, conducted using ImageJ IHC Profiler. n = 8 per group. Data are shown as the mean ± SD. Statistical significance was analyzed by one-way analysis of variance with the Bonferroni correction for multiple comparisons. *P < 0.05; **P < 0.01; ***P < 0.001; ns, not significant.

Our *in vivo* experiments supported the therapeutic potential of NDEFgel in wound healing. To investigate the underlying mechanisms of NDEFgel in depth, we applied transcriptomic analysis to the levels of typical regeneration-associated molecules by qRT-PCR. The standardized Z scores were normalized and visualized as a microarray heat map (**Figure 5A**). PCA of transcriptional results showed that the cluster of the NDEFgel treatment group segregated distinctively from the non-treatment groups than did the Prontosan treatment group, indicating that the gene expression of various regeneration-associated factors was systemically upregulated by NDEFgel therapy, which developed a more favorable pro-regenerative microenvironment (**Figure S15**). In addition, we found that the NDEFgel-treated group distinctly upregulated collagen I (Col1a1), collagen III (Col3a1), collagen II (Col12a1), MMP2, MMP9, PDGF-C, and IGF-1, within the wound microenvironment (**Figure 5A**), all of which are firmly associated with fibroblast behaviors 36, 39, 40. It is well accepted that fibroblasts, highly heterogeneous cells, generally transform into α-SMA-positive myofibroblasts to accelerate collagen deposition and wound closure 40. In this regard, Picrosirius-Red staining under a polarization microscope showed that in the NDEFgel-treated mice, the proportion and distribution of collagen fibrils were much closer to those of sham-operated mice than untreated and Prontosan-treated mice (**Figure 5B-C**). A higher proportion of collagen I and mixed collagen suggested that untreated and Prontosan-treated mice were still in the process of granulation proliferation (**Figure 5B-C**). By contrast, the altered proportion of collagen types in NDEFgel-treated mice provided an ideal collagen composition approaching the inherent composition in normal skin (**Figure 5B-C**), implying favorable potential of NDEFgel therapy in tissue remodeling and scar healing 37, 41. Collectively, these evidences make us hypothesize that the NDEFgel treatment involves the accelerated transformation of fibroblasts into α-SMA-positive myofibroblasts that facilitate collagen deposition and favor tissue remodeling.

To validate this hypothesis, we quantified the expression of α-SMA in repaired skin samples of mice after treatments. Indeed, our results indicated that NDEFgel treatment induced the highest α-SMA levels among all treatments (**Figure 5D**). Previously, it has been demonstrated that Rac1, a small GTPase in the Rho family expressed in fibroblasts, is required to modulate behaviors and functions of fibroblasts by manipulating cytoskeletal dynamics, thus contributing to wound healing 42, 43. We then measured Rac1 expression and indeed found a sharp increase in Rac1 levels in the NDEFgel-treated wound microenvironment (**Figure 5D**). Similar results were concluded from immunohistochemical analysis. Considerable α-SMA-positive myofibroblasts and Rac1 expression were widely distributed in skin from NDEF-treated mice and were significantly higher than those of untreated, Prontosan-treated and sham-operated mice (**Figure 5E-F**). Accordingly, we further hypothesized that NDEFgel promotes wound regeneration through controlled release of ND, which can modulate fibroblast behaviors to participate in healing process by a Rac1-dependent cytoskeletal rearrangement (**Figure S16**).

**Figure 6:**
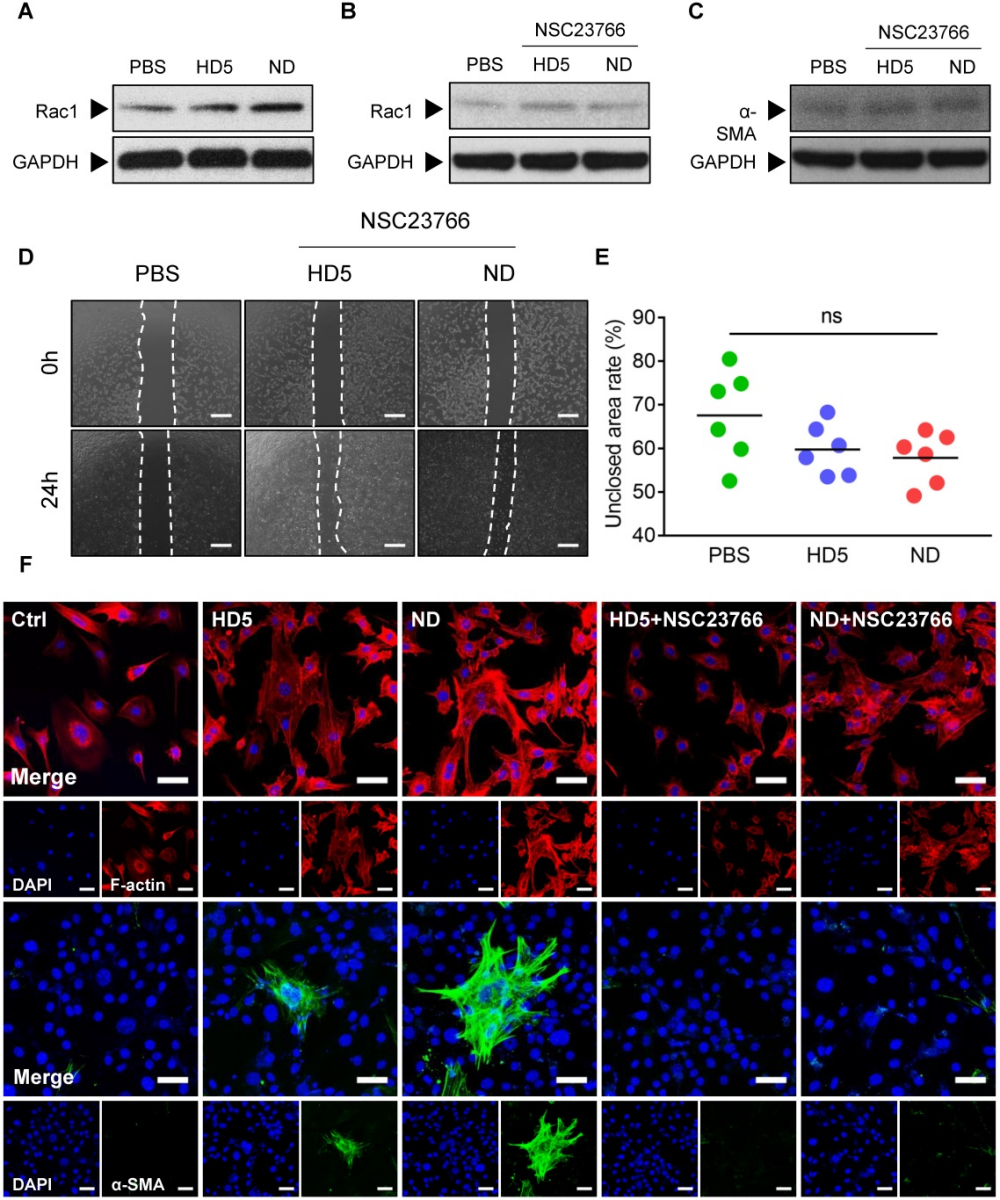
** ND modulated fibroblast behaviors through Rac-1-dependent cytoskeletal rearrangement.** (A) Western blotting detecting the expression levels of Rac1 in fibroblast 3T3 cells after stimulation with PBS, HD5 (12.5 µg/mL) or ND (12.5 µg/mL). GAPDH was adopted as an internal reference. Western blotting detection of the expression levels of Rac1 (B) and α-SMA (C) in fibroblast 3T3 cells after stimulation with PBS, HD5 (12.5 µg/mL) or ND (12.5 µg/mL) and pre-treatment with NSC23766 (50 µM). GAPDH was adopted as an internal reference. Representative images (D) and quantitative analysis (E) of fibroblast 3T3 migration upon the incorporation of PBS, HD5 (12.5 µg/mL) or ND (12.5 µg/mL) with pre-treatment with NSC23766 (50 µM), determined using an *in vitro* wound scratch assay. Scale bars, 1 mm. n = 6 per group. (F) Fluorescence images of F-actin and α-SMA in fibroblast 3T3 cells after stimulation with PBS, HD5 (12.5 µg/mL) or ND (12.5 µg/mL) with/without pre-treatment with NSC23766 (50 µM). Red, F-actin; Green, α-SMA; Blue, DAPI. Scale bars, 50 µm. Data are shown as the mean ± SD. Statistical significance was analyzed by one-way analysis of variance with the Bonferroni correction for multiple comparisons. *P < 0.05; **P < 0.01; ***P < 0.001; ns, not significant.

To validate our hypothesis, we initially monitored alterations in Rac1 levels in fibroblast 3T3 after stimulation with ND/HD5 *in vitro*. As expected, we observed remarkably elevated expression of Rac1 in fibroblasts treated with ND, and strikingly, ND treatment enhanced higher Rac1 levels than HD5 stimulation (**Figure 6A and Figure S17**). Therefore, we wondered whether Rac1 is an indispensable regulator involved in ND-induced alterations of fibroblast behaviors. Consequently, we used NSC23766 to limit the expression and activation of Rac1 before treatment with ND. As expected, pre-treatment with NSC23766 completely abolished the increase in Rac1 induced by ND (**Figure 6B and Figure S18**). We found that pre-treatment with NSC23766 abolished the aberrantly high expression of α-SMA in ND-treated fibroblasts (**Figure 6C and Figure S19**). Additionally, a wound scratch assay demonstrated that the Rac1 inhibitor attenuated the ability of ND to promote fibroblast migration (**Figure 6D-E**). The above factors collectively suggested that ND regulated the migration and differentiation of fibroblasts in a Rac1-dependent manner. In terms of the inherent ability of Rac1 to modulate cytoskeletal dynamics, we next characterized cytoskeletal rearrangement via fluorescence staining of F-actin. We observed that ND-stimulated fibroblasts showed a membrane-extended cell shape with more pseudopodia, including lamellipodia (**Figure 6F**), in which the redistribution of F-actin characterized a cytoskeletal rearrangement contributing to cell migration (**Figure 6F**). Although the HD5-stimulated fibroblasts also displayed actin rearrangement, the effect was much weaker than that of ND (**Figure 6F**). Moreover, we found that specific inhibition of Rac1 restricted cytoskeletal rearrangement and ND/HD5-induced cell shape changes (**Figure 6F**). This phenomenon was concordant with the alterations in fibroblast migration induced by ND. Furthermore, we also investigated α-SMA morphology. Without ND stimulation, fibroblasts seldom expressed α-SMA, indicating that most fibroblasts remained in a quiescent state without transforming into myofibroblasts (**Figure 6F**). Upon treatment with HD5, few α-SMA-positive myofibroblasts appeared, with a sparse α-SMA network surrounding the nucleus (**Figure 6F**). Remarkably, after stimulation with ND, a significant proportion of primary fibroblasts transformed into α-SMA-positive fibroblasts, whose α-SMA skeleton cross-assembled into a dense and regular network with sufficiently extended connections between all positive cells (**Figure 6F**). In addition, pre-treatment with NSC23766 completely abolished the expression and skeletal alterations of α-SMA mediated by ND and HD5 (**Figure 6F**). In summary, we proved that the mode of action of NDEFgel in skin-repair is due to the controlled release of ND, which acts upon fibroblasts to promote their migration and accelerate α-SMA-positive myofibroblast transformation in a Rac1-dependent manner (**Figure S16**).

## Discussion

Here, we demonstrated that ND with hydrogel delivery could effectively accelerate wound healing in full-thickness excisional murine wound models by exhibiting both antimicrobial and pro-regenerative functions. We further showed that the pro-regenerative effect was mediated by the improved behaviors of fibroblasts and subsequent collagen deposition, which might contribute to favorable tissue remodeling. Additional evidence showed that ND could promote the migration of fibroblasts and differentiation to myofibroblasts, through Rac1-dependent cytoskeletal rearrangement.

Owing to the complexity of the wound microenvironment (*e.g.,* abundant proteases and inflammatory conditions) 39, a proper carrier is required for local usage of ND to improve stability and pharmacodynamics. Due to favorable biocompatibility, biodegradability, and controlled release, hydrogels have contributed widespread applications to various fields, including tissue engineering and drug delivery 44. However, hydrogel platforms based on different gelation mechanisms such as hydrophobic assembly, ionic gelation, and thermocondensation would have different effects on the function and activity of cargo agents 9, 44. Consequently, although hydrogel delivery has already been considered a reliable local platform to favor the controlled release of cargo drugs 44, its feasibility in delivering HDPs should be carefully evaluated. Puthia et al. found that hydrogel delivery using carboxymethyl cellulose (CMC) or Plu significantly diminished the antimicrobial/anti-endotoxin activity of TCP-25 by affecting secondary structures, while TCP-25 could maintain the correct conformation in hydroxypropyl cellulose (HPC) hydrogel 9. In this study, Alg gel significantly diminished ND activity, which possibly be due in part to the alterations of β-sheets in the presence of Alg gel, because of the critical roles of correct β-folding in the antimicrobial activity of defensins 19. In this regard, we speculate that the presence of cationic ions (Ca^2+^ and Na^+^) in Alg hydrogels possibly disrupts salt bridges within the peptide backbone, which might result in conformational changes in β-sheets causing inhibition of activity 15, 19; of course, this speculation needs future validation. Regarding the unfavorable influence of TG18 on ND, we supposed that flexible fatty chains of TG18 may interfere with nanostructural formation via hydrophobic interactions with myristoyl chains of ND, thereby limiting the activity, because we previously found that adding DSPE-PEG (a surfactant containing flexible fatty chain) into ND largely attenuated ND activity through a potential influence on nanoassembly 20. Consequently, rational selection of pharmacologically acceptable carriers is very important when formulating HDPs-related therapeutics, which will determine the eventual therapeutic performance. According to the results of activity testing as well as *in vitro* characterization of degradation, drug release, and antimicrobial kinetics, the optimal carrier favoring ND delivery among tested hydrogels was finally determined to be the Plu hydrogel which has already been applied in the controlled delivery of diverse agents, such as small molecules 45, genes 33, and even bacteria 46, due to its thermosensitive nature and *in situ* assembly behaviors as well as excellent biocompatibility.

Wound healing is a complex multistep process involving blood clot formation, re-epithelialization, granulation proliferation, inflammation, neovascularization, collagen deposition and remodeling 39, 40. It was clearly observed that the application of NDEFgel greatly benefited the whole healing process, particularly collagen deposition. We reported that fibroblasts, as effector cells, might be involved in NDEFgel-induced pro-regenerative roles, because we found a sharp increase in the expression of genes related to fibroblast behaviors. Collagen I-III, which are produced by fibroblasts, are the most abundant collagen subtypes in the skin 36, 40. MMP2 and MMP9 usually participate in collagen remodeling 39. PDGF-C and IGF-1 signaling contribute to myofibroblast heterogeneity 36. Our* in vitro* assays indeed showed that ND could improve migration of fibroblasts and subsequent transformation to myofibroblasts, which possibly contribute to superior collagen deposition and remodeling in NDEFgel-treated mice 47. As expected, we observed the NDEFgel-treated wounds accumulated more myofibroblasts (α-SMA-positive fibroblasts). Fibroblasts/myofibroblasts can secrete various growth factors, such as epidermal growth factor (EGF) 3. Thus, increased fibroblasts in NDEFgel-treated wounds might be one of the possible mechanisms leading to the enhanced activation of the EGFR pathway; of course, it is possible that ND directly activates EGFR signaling and future efforts are needed for validation. The ability of ND to manipulate the behaviors of functional cells inspires us to explore additional therapeutic molecules that could regulate the behaviors of functional cells, thus pushing us to establish multifunctional/multimodal approaches for personalized wound therapy. For example, incorporation of hepcidin into our NDEFgel might achieve a more balanced pro-regenerative microenvironment by manipulating the behaviors of both fibroblasts and neutrophils in cutaneous injury.

In our study, we used a therapeutic dose of NDEFgel that scaled up to 1/20 of the therapeutic dose of active ingredients (0.1%) in Prontosan hydrogel 48, but achieved significantly higher therapeutic efficacy than Prontosan. Prontosan indeed decreased the bacterial burden in wounds, but its contribution to boosting skin regeneration and healing was poor, which might result from its tissue toxicity and inability to promote regeneration. Future work should explore the depth and breadth of the efficacy of NDEFgel, especially comparisons between NDEFgel and Prontosan treatments in clinical trials, to determine the scope of adaptive treatments with NDEFgel. We envision the implementation of a phase 1 trial to identify an optimal dosing regimen for NDEFgel in patients, which is necessary because of the high costs of ND synthesis currently, and thus the optimal bioavailability plays a significant role in future clinical translation. As the costs of peptide synthesis, recombinant protein expression and hydrogel manufacturing continue to decrease, ultimately, these trends will advocate for future research into the utility of novel ND-containing hydrogels for rapid and generical treatment of life-threatening complications in wound-healing.

Limitations of this study include the lack of real-time *in vivo* investigation of ND release behaviors, hydrogel degradation, and neonatal skin formation, which can reveal the pharmacokinetic and pharmacodynamic properties of our hydrogel formulation to help design clinical trials and drive translation 9. Besides, this study did not systemically uncover the cell kinetics of the pro-regenerative microenvironment developed by NDEFgel at the single-cell level, which will help identify therapeutic mechanisms of engineering biomaterials in more depth in the future. Furthermore, this study mainly focuses on murine models whose wound contraction leads to accelerated wound closure 49. More translational value will be presented by adopting humanized murine models 30, 41, big animal models (*e.g.,* porcine models) and even human trials; besides, adopting wound models with a more extended infectious time (*e.g.,* 24 h) to evaluate therapeutic performance of our formulations might be more clinically adaptive, which will be carefully tested in our future investigation.

In summary, our findings demonstrate the dual action of ND that eradicates infection and modulates fibroblast behaviors. We successfully engineered ND into a practical hydrogel dressing with distinct therapeutic efficacy in full-thickness excisional murine models with MRSA infection, which holds promising potential for advanced wound management in the clinic. In the future, we will continue to accelerate the clinical translation of our ND and NDEFgel.

## Supplementary Material

Supplementary figures and tables.

## Abbreviations

HDP(s): host defense peptides
ND: nanodefensin
HD5: human α-defensin 5
NDEFgel: nanodefensin-encased hydrogel
SPPS: solid-phase peptide synthesis
CD: circular dichroism
SEM: scanning electron microscopy
TEM: transmission electronic microscopy
Plu: pluronic F127
HBTU: (1-H-benzotriazol-1-y1)-1,1,3,3-tetramethyluronium hexafluorophosphate
DIEA: diisopropylethylamine
RP-HPLC: reverse-phase high-performance liquid chromatography
ESI-MS: electrospray ionization mass spectrometry
TG18: triglycerol monostearate
Alg: alginate
DLS: dynamic light scattering
MRSA: methicillin-resistant *Staphylococcus aureus*
LB: Luria-Bertani
ddH_2_O: double-distilled water
H&E: hematoxylin and eosin
α-SMA: α-smooth muscle actin
GRAS: generally recognized as safe
G': storage modulus
G”: loss modulus
EGFR: epidermal growth factor receptor
PCA: principle component analysis
F-actin: filamentous actin
