# Supplementary material for: Nanodefensin-encased hydrogel with dual bactericidal and pro-regenerative functions for advanced wound therapy
Source: Theranostics. 2021 Jan 26;11(8):3642–60. doi: 10.7150/thno.53089 (PMC7914350; doi:10.7150/thno.53089)
Supplement: Supplementary file 1 — Supplementary figures and tables. [file thnov11p3642s1.pdf]

Supplementary Materials

Nanodefensin-encased hydrogel with dual bactericidal and pro-regenerative functions for advanced wound therapy

Gan Luo,[1]† Yaqi Sun,[1]† Jue Zhang,[1] Zhipeng Xu,[1] Wuyuan Lu,[4] Hanbin Wang,[1] Yan Zhang,[1] Hui Li,[1] Zhengwei Mao,[2] Shixin Ye,[3]* Baoli Cheng,[1]* Xiangming Fang[1]*

[1]Department of Anesthesiology and Intensive Care, The First Affiliated Hospital, School of Medicine, Zhejiang University, Hangzhou 310003, China.

[2]Department of Polymer Science and Engineering, Zhejiang University, Hangzhou 310027, China.

[3]Laboratory of Computational and Quantitative Biology (LCQB), Institute of Biology, Paris-Seine, Sorbonne University, Paris, 75006, France.

[4]Key Laboratory of Medical Molecular Virology (MOE/NHC/CAMS), School of Basic Medical Sciences, Fudan University, Shanghai, 200032, China.

*Corresponding Authors: X. Fang (xmfang@zju.edu.cn); B. Cheng (chengbaoli1979@zju.edu.cn); Shixin Ye: (shixin.ye-lehmann@inserm.fr).

†G. L. and Y. S. contributed equally to this work.

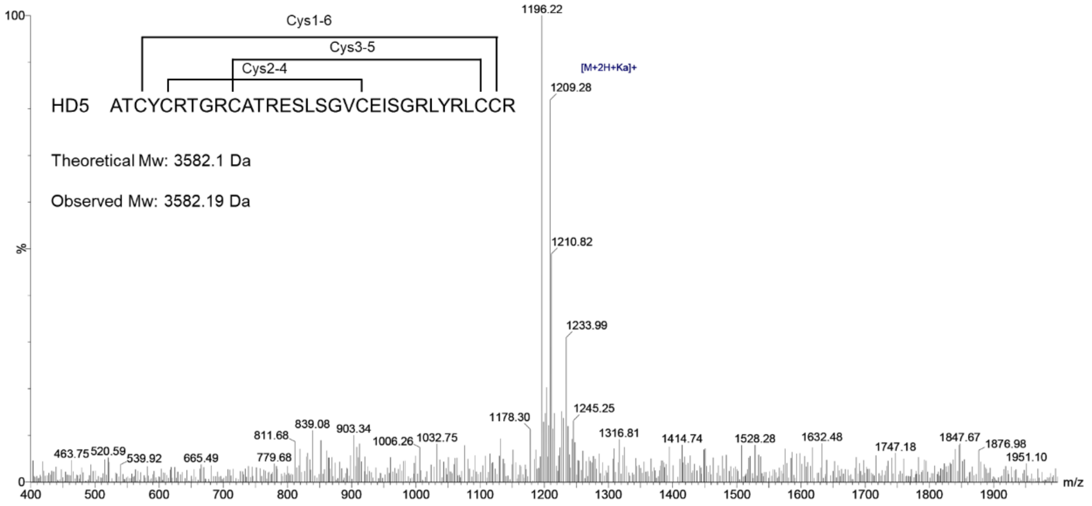

Figure S1. Electrospray ionization mass (ESI-MS) analysis of HD5. The mass-to-charge (m/z) is [M+2H]/3. Therefore the observed molecular weight is 3582.19 Da, in compared to the theoretical molecular weight of 3582.1 Da.

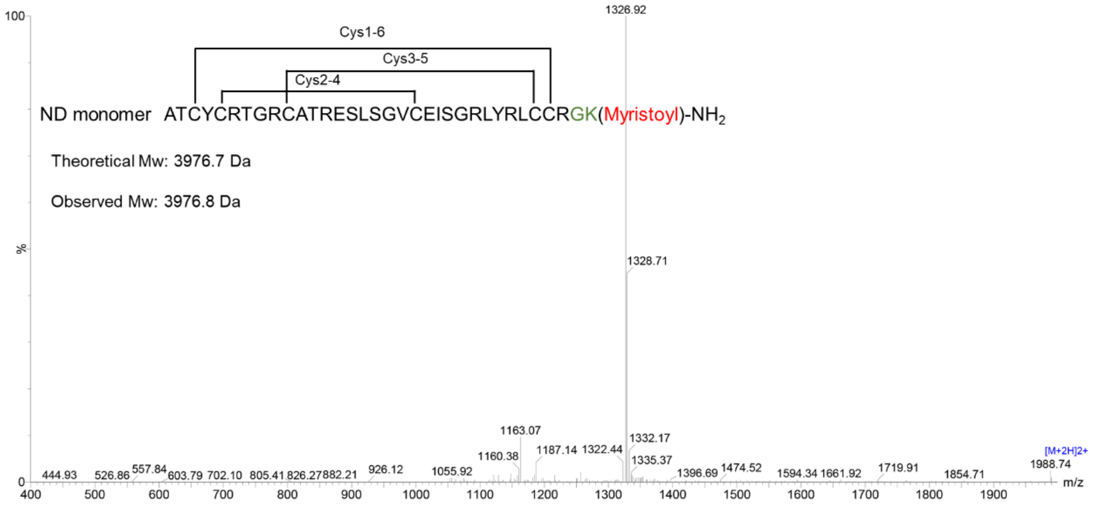

Figure S2. ESI-MS analysis of myristoylated HD5 (ND monomer). The mass-to-charge (m/z) is [M+H]/3. Therefore, the observed molecular weight is 3976.8 Da, in compared to the theoretical molecular weight of 3976.7 Da.

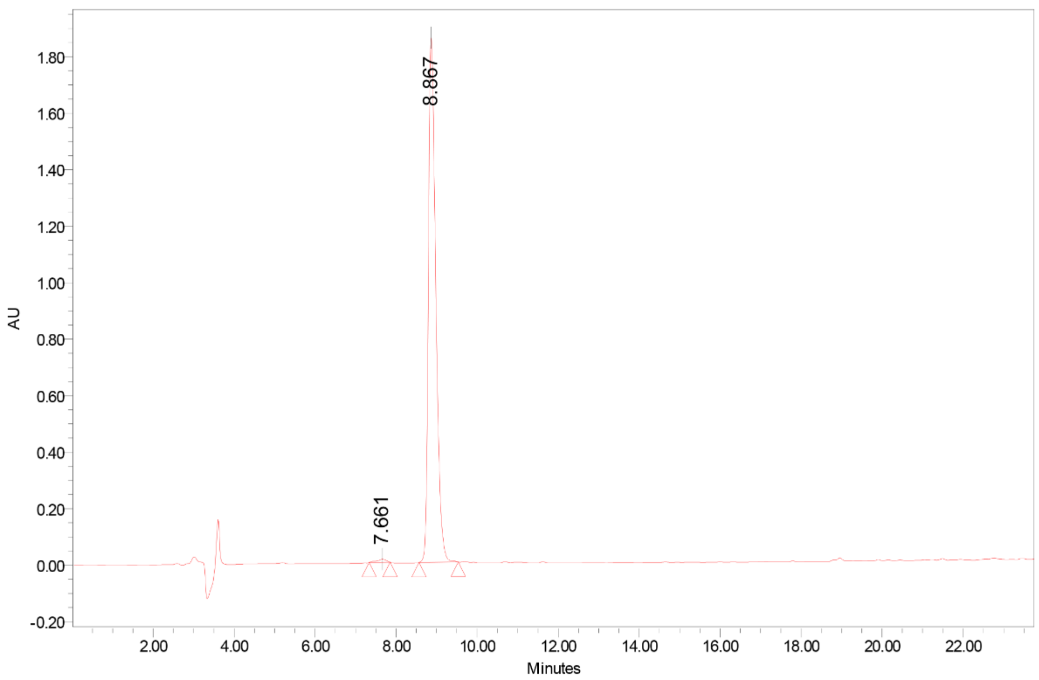

Figure S3. Representative chromatogram of HD5 analyzed by reverse-phase (RP) high-performance liquid chromatography (HPLC). The numbers indicate the retention times of HD5 (8.867 min) and the impurity (7.661 min).

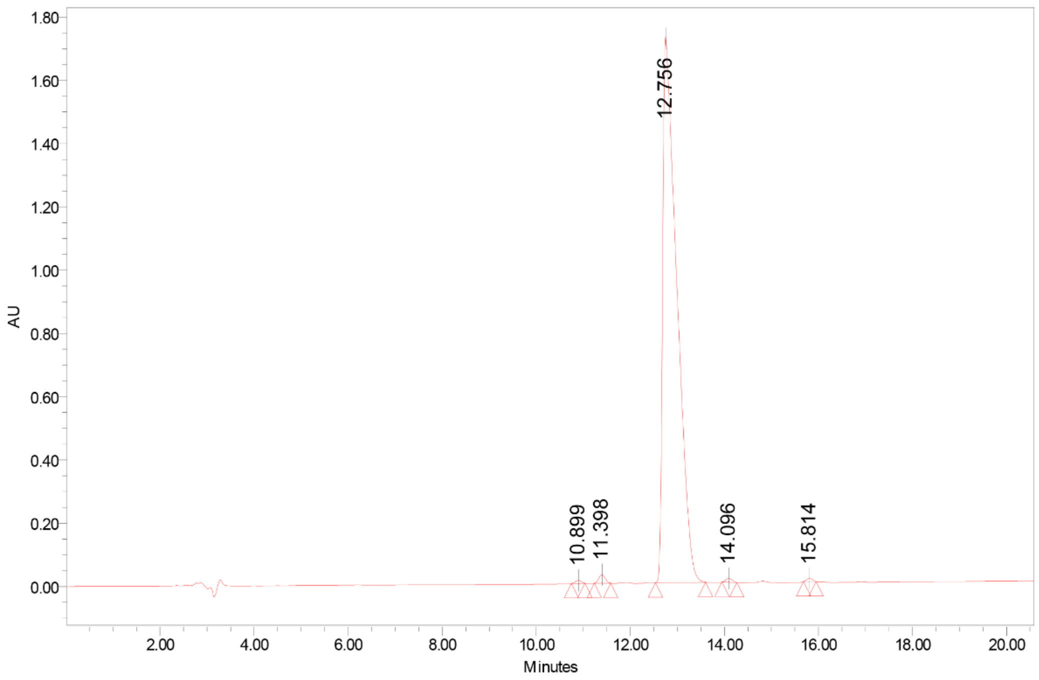

Figure S4. Representative chromatogram of ND monomer analyzed by HPLC. The numbers indicate the retention times of ND monomer (12.756 min) and several impurities (10.899, 11.398, 14.096, and 15.814 min).

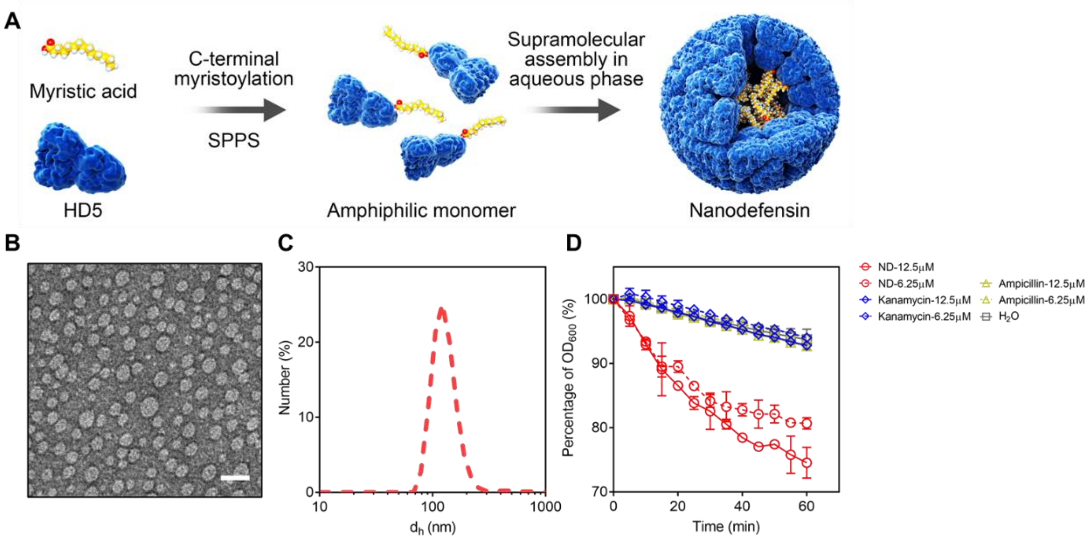

Figure S5. (A) Schematic diagram illustrating the molecular reprogramming and supramolecular assembly of ND. (B) Representative TEM image of the supramolecular nanostructures of ND in water. Scale bars, 100 nm. (C) Number distribution of hydrodynamic diameter (dh) for ND. (D) Antimicrobial activity of ND against MRSA compared with the commercial antibiotics kanamycin and ampicillin. The bacterial suspension ($OD_{600} \approx 0.4$) was incubated with ND (6.25 μM or 12.5 μM), kanamycin (6.25 μM or 12.5 μM), ampicillin (6.25 μM or 12.5 μM), or sterile water and the OD_{600} was dynamically monitored every 5 min for 1 h. n = 2 per time point per group.

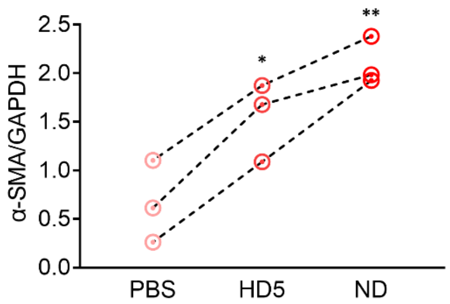

Figure S6. Quantification of expression levels of α-SMA in fibroblast 3T3 cells. Cells were stimulated by PBS, HD5 (12.5 μg/mL), or ND (12.5 μg/mL). Values represent the mean ± SD of three biological replicates (n = 3). GAPDH was adopted as an internal reference. $^{*}P < 0.05$, $^{**}P < 0.01$, $^{***}P < 0.001$.

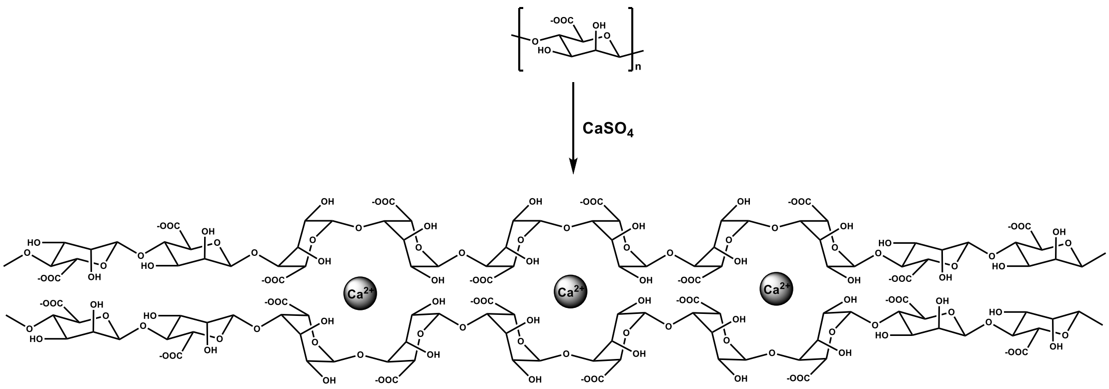

Figure S7. The chemical mechanism of ionic gelation of alginate hydrogel.

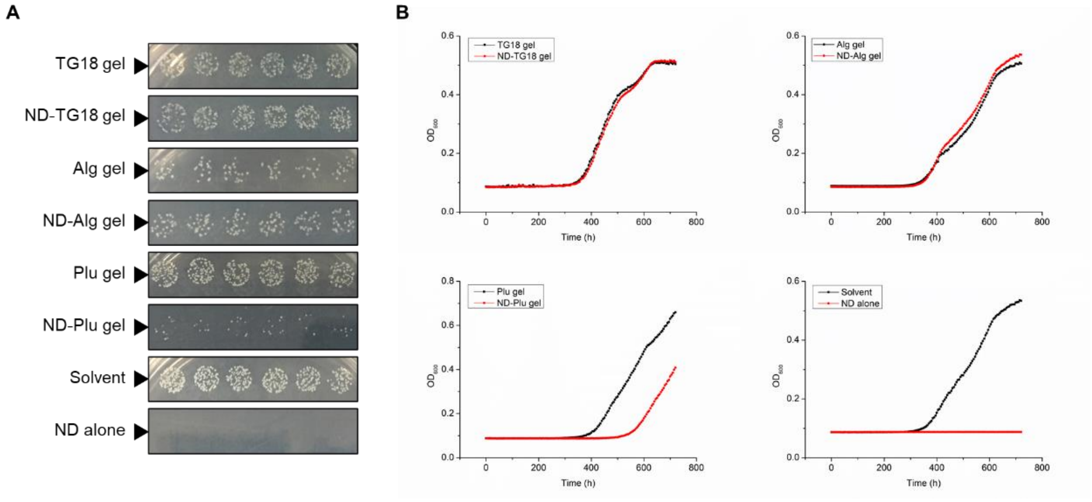

Figure S8. (A) Photographic images of viable count measurements of living MRSA after fully mixing with various hydrogel formulations. (B) The growth curves of MRSA suspension after fully mixing with various hydrogel formulations.

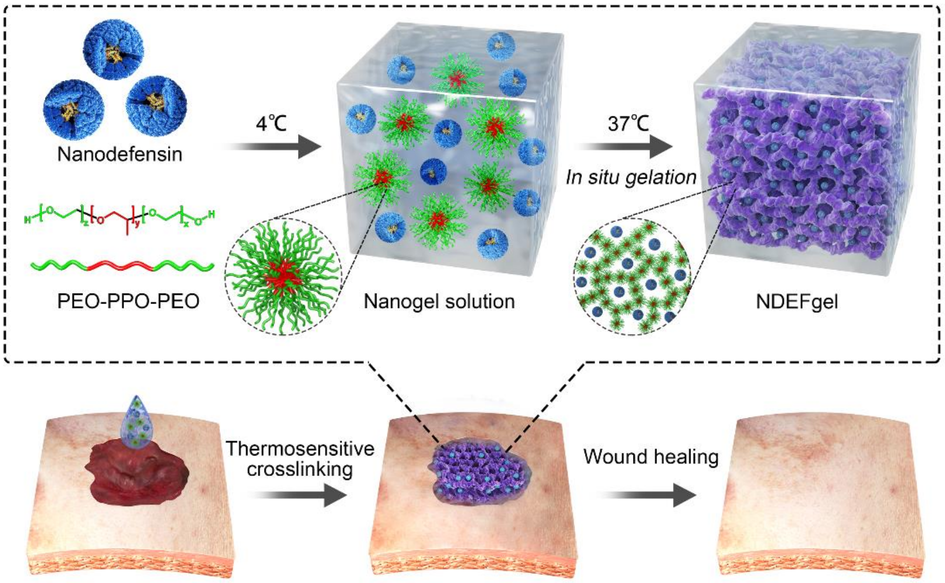

Figure S9. Schematic diagram illustrating that ND delivered by a hydrogel platform based on Plu effectively assists wound regeneration.

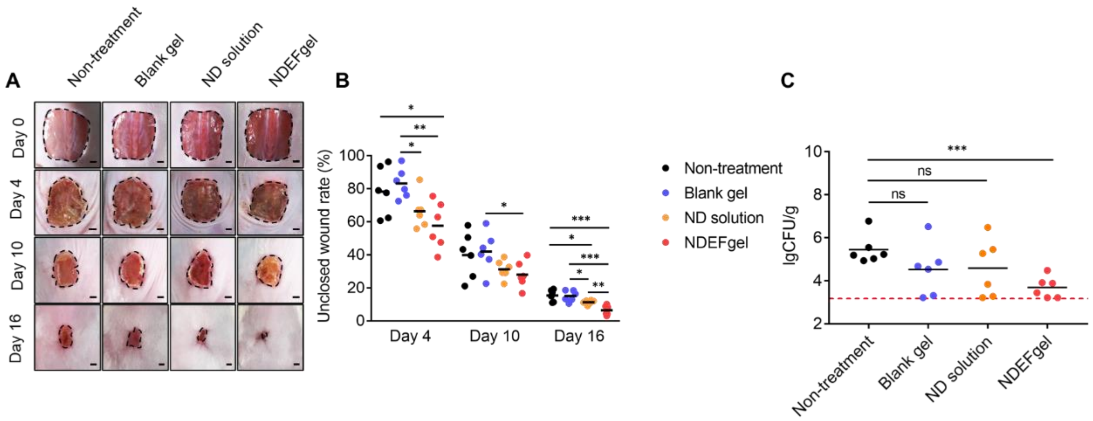

Figure S10. Therapeutic efficacy of NDEFgel on murine wound models complicated with MRSA infection, compared with that of non-treatment, blank gel, and ND solution. (A) Macroscopic imaging of wounds immediately following surgery (Day 0) and after 4, 10, or 16 days of treatment with different interventions including non-treatment, blank gel, ND solution, and NDEFgel. Scale bars, 2 mm. (B) Quantification of unclosed wound rates in the murine wound models after 16 days of treatment. n = 6 per group. (C) Determination of cutaneous bacterial burden in murine wound models treated with different formulations. Dashed line indicates the limit of detection. **P* < 0.05; ***P* < 0.01; ****P* < 0.001; *****P* < 0.0001; ns, not significant.

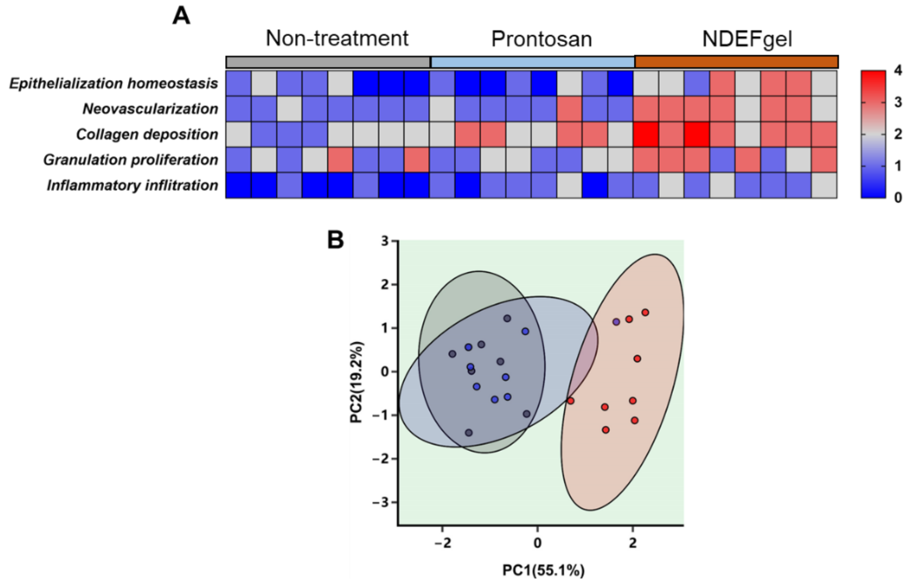

Figure S11. (A) Histopathological scores of skin tissues from murine wound models according to results of H&E staining and Masson's trichrome staining to evaluate different aspects of wound healing including epithelialization homeostasis, neovascularization, collagen deposition, granulation proliferation, and inflammatory infiltration. n = 8 per group. (B) Principal component analysis (PCA) of skin scores

demonstrated that the cluster of the NDEFgel treatment group (red region) was separated from the clusters of all other groups, and that overlap was seen between Prontosan (blue region) and the non-treatment groups (grey region).

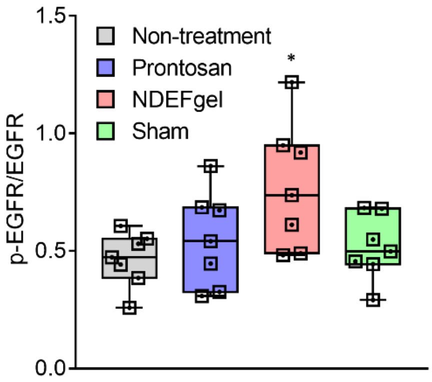

Figure S12. Protein levels of p-EGFR and EGFR in skin tissues. The ratios of p-EGFR/EGFR were analyzed for different conditions including Non-treatment, Prontosan, NDEFgel, and Sham-operated treatment. Data are shown as means ± SD of seven biological replicates (n = 7). **P* < 0.05; ***P* < 0.01; ****P* < 0.001.

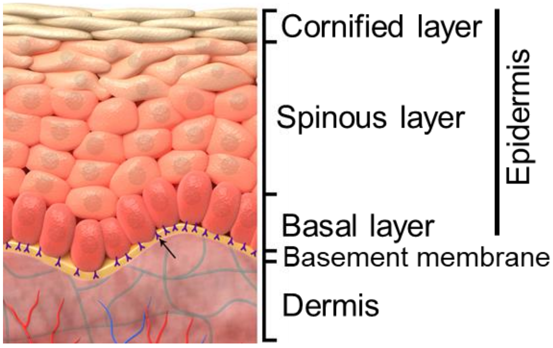

Figure S13. Schematic diagram illustrating the cutaneous structure.

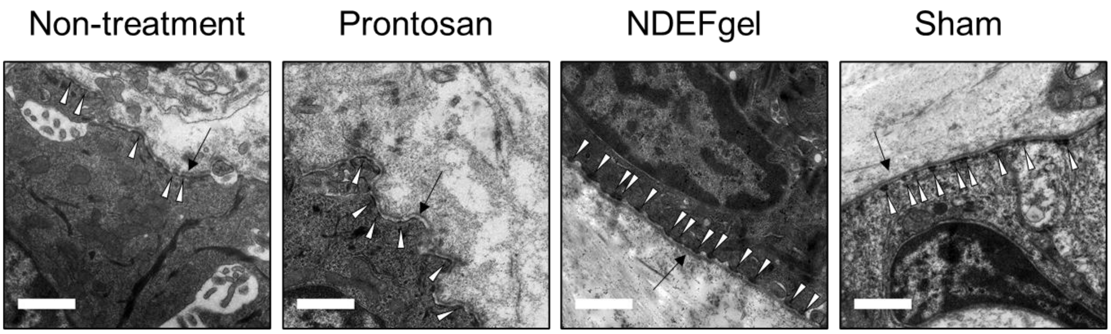

Figure S14. TEM characterization of skin tissues. The morphology of non-treated skin tissues or treated with Prontosan, NDEFgel, and Sham-operation. Black arrows indicate the basement membrane of skin tissues. White arrows indicate the arrangements of hemidesmosomes attached to the basement membrane. Scale bars is 1 μm.

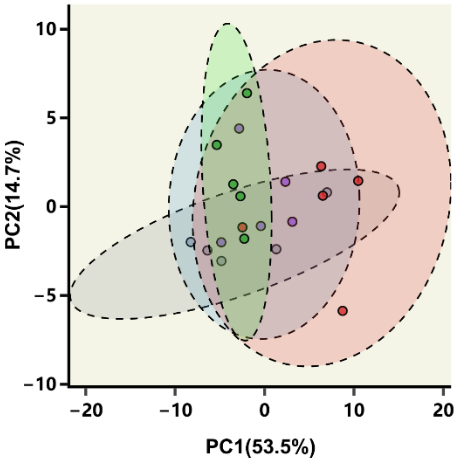

Figure S15. PCA of the transcriptome in the wound microenvironment demonstrated that the cluster of the NDEFgel treatment group was the most clearly segregated from the other groups. The red region, NDEFgel group; blue region, Prontosan group; grey region, non-treatment group; green region, sham-operated group.

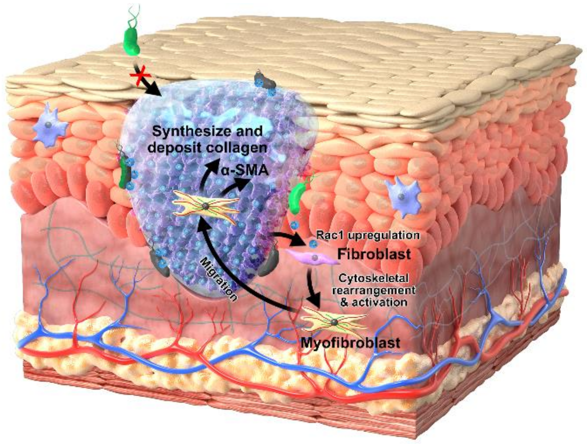

Figure S16. Schematic diagram illustrating the possible mechanism of NDEFgel effective management of wound regeneration. 3D diagram of the epidermis can be divided into three parts from top to bottom: the cornified region (yellow), the spinous (orange) and basal (red) layers. The subcutaneous layer with adipose cells, blood vessels and muscles cells were also depicted. NDEFgel (semi-transparent globular structure) releases ND monomer (blue balls) that upregulates Rac1 in fibroblast cells (pink). Besides, fibroblasts, highly heterogeneous cells, generally transform into α-SMA-positive myofibroblasts to accelerate collagen deposition and wound closure. ND monomer as an antimicrobial polypeptide attaches to the bacterial (green) membrane to exhibit bactericidal activity.

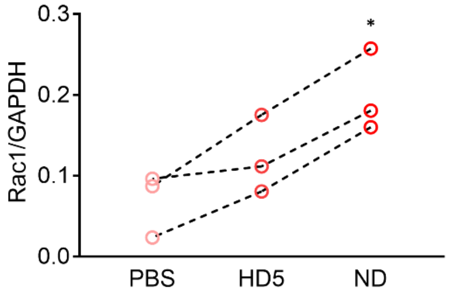

Figure S17. Quantification of expression levels of Rac1. Fibroblast 3T3 cells after stimulated by PBS, HD5 (12.5 μg/mL) or ND (12.5 μg/mL) were assayed with Rac1 expression. GAPDH was adopted as internal reference. *$P < 0.05$; **$P < 0.01$; ***$P < 0.001$.

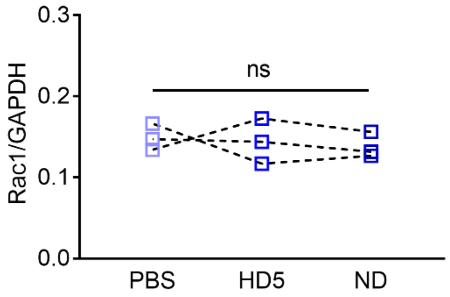

Figure S18. Quantification of expression levels of Rac1 in the presence of NSC23766. Fibroblast 3T3 cells after stimulated by PBS, HD5 (12.5 μg/mL) or ND (12.5 μg/mL) with pre-treatment of NSC23766 (50 μM), a selective inhibitor of Rac1, were assayed with Rac1 expression. GAPDH was adopted as internal reference. *$P < 0.05$; **$P < 0.01$; ***$P < 0.001$.

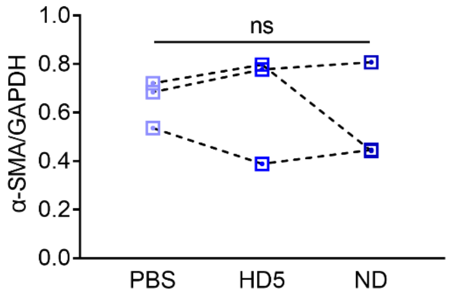

Figure S19. Quantification of expression levels of α-SMA in the presence of NSC23766. Fibroblast 3T3 cells after stimulated by PBS, HD5 (12.5 μg/mL) or ND (12.5 μg/mL) with pre-treatment of NSC23766 (50 μM), a selective inhibitor of Rac1, were assayed with α-SMA expression. GAPDH was adopted as an internal reference. *$P < 0.05$; **$P < 0.01$; ***$P < 0.001$.

Table S1. Mouse primers used for gene expression quantification.

Gene	Forward primer (5'-3')	Reverse primer (5'-3')
HGF	ATGTGGGGGACCAAACTTCTG	GGATGGCGACATGAAGCAG
FGF7	CTCTACAGGTCATGCTTCCACC	ACAGAACAGTCTTCTCACCCT
FGF10	TTTGGTGTCTTCGTTCCCTGT	TAGCTCCGCACATGCCTTC
HB-EGF	CGGGGAGTGCAGATACCTG	TTCTCCACTGGTAGAGTCAGC
TGF-α	CACTCTGGGTACGTGGGTG	CACAGGTGATAATGAGGACAGC
TGF-β1	ACGCCTGAGTGGCTGTCTTTTGAC	GGGCTGATCCCGTTGATTTCCACG
VEGFA	CGACACGGGAGACAATGGGATGAA	AGGGGCGGGGTGCTTTTGTAGACT
FGF2	GCGACCCACACGTCAAACTA	TCCCTTGATAGACACAACTCCTC
MMP-2	CTTCGCTCGTTTCCTTCAAC	ATGTCAGACAACCCGAGTCC
MMP-9	ATCCCCAGAGCGTCATTCGCG	CACGTAGCCCACGTCGTCCAC
PDGF-α	CGCTGCACTGGCTGTTGTAA	GCCGGCTCATCTCCACCTCA
PDGF-C	GGAACAGAACGGAGTGCAAGA	TGAGGAAACTTCGGGCTGTG
IGF-1	GCCCAAGACTCAGAAGTCCCCG	TGCAGGTTGCTCAAGCAGCA
Col1a1	ACGTCCTGGTGAAGTTGGTC	TCCAGCAATACCCTGAGGTC
Col3a1	CTGGTCCTGTTGGTCCATCT	ACCTTTGTCACCTCGTGGAC
Col12a1	GCTATCCAGGTTCCGGCTAA	CCCTCCTGTATGATGCCGAC
IL-1α	TTGGTTAAATGACCTGCAACA	GAGCGCTCACGAACAGTTG
IL-1β	TTGACGGACCCCAAAAGAT	GAAGCTGGATGCTCTCATCTG
IL-6	ACCGCTATGAAGTTCCTCTCTGCAA	TGCAGGTTGCTCAAGCAGCA
MCP-1	CAACTCTCACTGAAGCCA	CAGCACAGACCTCTCTCTT
TNF-α	CTGAACTTCGGGGTGATCGG	GGCTTGTCACTCGAATTTTGAGA
